# Predicting short-term composite outcome risk in heart failure patients using a machine learning model incorporating UHR: a retrospective cohort study

**DOI:** 10.3389/fnut.2026.1846081

**Published:** 2026-06-01

**Authors:** Yihe Zhang, Xu Zhang, Hongbin Song, Qiyu Hu, Jingzhu Nan

**Affiliations:** 1 Department of Clinical Laboratory, Beijing Anzhen Hospital, Capital Medical University, Beijing, China; 2 Department of Clinical Laboratory, The First Medical Center of Chinese PLA, Beijing, China; 3 Department of Clinical Laboratory, The Third Medical Center of Chinese PLA, Beijing, China

**Keywords:** heartfailure, interpretable machine learning, machine learning, prognosis, uric acid-to-high-density lipoprotein cholesterol ratio

## Abstract

**Introduction:**

The uric acid-to-high-density lipoprotein cholesterol ratio (UHR) is a composite biomarker that reflects both oxidative stress and antioxidant imbalance. Previous studies have linked UHR to poor prognosis in heart failure (HF), but the evidence relies largely on traditional regression models; it lacks independent validation and has not been integrated into modern, individualized risk-prediction tools. This study aimed to independently validate the association between UHR and a 180-day composite outcome (all-cause death or HF rehospitalization) in a relatively large HF cohort, and to construct an interpretable machine learning model that incorporates UHR and routine clinical indicators.

**Methods:**

This was a single-center retrospective cohort study that consecutively enrolled 2,737 patients with heart failure hospitalized at Beijing Anzhen Hospital, Capital Medical University, from January 1, 2024, to June 30, 2025. All patients were followed up until December 31, 2025, to ensure complete 180-day follow-up data for each patient. The primary outcome was a composite endpoint of all-cause death or heart failure rehospitalization within 180 days. Multivariate Cox regression and restricted cubic splines (RCS) were used to analyze the association and dose–response relationship between UHR and the composite outcome. Least Absolute Shrinkage and Selection Operator (LASSO) regression combined with multivariate Logistic regression was employed to screen predictive variables. Seven machine learning models were developed in the training set (*n* = 1,917) and validated in an internal validation set (*n* = 820). The optimal model was interpreted using the SHapley Additive exPlanations (SHAP) framework and deployed as an online calculator.

**Results:**

During follow-up, 926 patients (33.8%) experienced the composite outcome. UHR was confirmed as an independent risk factor for the composite outcome. RCS analysis revealed a nonlinear association. The eXtreme Gradient Boosting (XGBoost) model, constructed based on six variables: body mass index (BMI), history of hyperlipidemia, homocysteine (Hcy), glycated albumin (GA), small dense low-density lipoprotein cholesterol (sdLDL-C) and UHR, demonstrated the best performance. In the internal validation set, it achieved an AUC of 0.866 (95%CI, 0.839–0.890), with good calibration and high clinical net benefit in decision curve analysis. SHAP analysis identified sdLDL-C and UHR as the two most important predictive features and revealed nonlinear effects of the variables. The model was deployed as an online calculator (https://machine11.shinyapps.io/xgboostforUHR/), which currently serves as a research demonstration platform and requires external validation before any clinical application.

**Discussion:**

UHR is an independent risk factor for the 180-day composite outcome in HF patients. The XGBoost model based on UHR and five other routine clinical indicators shows good discrimination, calibration, and clinical net benefit. The online calculator provides an exploratory tool for short-term risk stratification but requires further external validation before clinical use.

## 1 Introduction

Heart failure (HF) is a major public health challenge in the field of cardiovascular medicine in the 21st century (1, 2). It affects approximately 64.3 million people worldwide, and its prevalence continues to rise due to the aging population (3, 4). Despite continuous advancements in diagnostic and therapeutic techniques, the prognosis for patients with heart failure remains poor, with a five-year mortality rate as high as 50%. Recurrent hospitalizations due to worsening heart failure impose a substantial burden on both patients and healthcare systems (5, 6). Therefore, accurately identifying high-risk individuals and implementing early interventions in clinical practice are crucial for improving outcomes in patients with HF and optimizing the allocation of medical resources.

Heart failure is a multisystem syndrome involving hemodynamic disturbances, neuroendocrine activation, metabolic disorders, and immune-inflammatory responses (7, 8). In recent years, composite biomarkers that integrate information from different pathophysiological pathways have gained attention for their ability to surpass traditional risk factors (9). For example, the triglyceride-glucose index (TyG) has been shown to have a long-term trajectory closely associated with the risk of adverse events in elderly patients with HF (10). The neutrophil percentage-to-albumin ratio, reflecting inflammatory-nutritional imbalance, has demonstrated good prognostic value in chronic HF (11). These studies collectively suggest that assessing patient status from a multidimensional perspective may more accurately capture the complex nature of HF progression than relying on a single biomarker.

The uric acid-to-high-density lipoprotein cholesterol ratio (UHR) is a recently emerged composite indicator (12, 13). Uric acid (UA), the end product of purine metabolism, is produced in increased amounts under conditions of hypoxia and inflammation and can exacerbate myocardial injury by activating the NLRP3 inflammasome (14). High-density lipoprotein cholesterol (HDL-C) exerts cardiovascular protective functions through its antioxidant and anti-inflammatory functions (15, 16). Therefore, UHR integrates these two opposing pathways and may more comprehensively reflect the balance of the metabolic-inflammatory network in patients with HF (17). Several studies have already provided preliminary evidence of the significance of this ratio in cardiovascular disease (13, 18). However, existing evidence is mostly based on traditional regression models, which are insufficient to capture the complex nonlinear interactions between UHR and other clinical variables, nor have they effectively integrated UHR into an individualized prediction tool convenient for clinical use.

Traditional logistic regression-based nomograms remain widely used in clinical practice for their simplicity and ease of bedside use (19). However, these models typically rely on linearity assumptions and may inadequately capture the complex, nonlinear interactions among clinical variables that characterize heart failure progression (20, 21). Moreover, the key value of any novel biomarker or prediction model lies not merely in its standalone performance, but in its incremental value-the ability to improve risk discrimination beyond that provided by established clinical variables (22). Based on this, our study is the first to introduce UHR as a core predictor into a machine learning (ML) framework. We aim to systematically evaluate the independent relation and dose–response relationship between baseline UHR levels and the 180-day composite outcome of all-cause death or heart failure rehospitalization. On this basis, we will combine UHR with routine clinical indicators to construct an interpretable risk prediction model. The decision-making logic of the model will be revealed using the SHapley Additive exPlanations (SHAP) framework to enhance clinical trustworthiness. Finally, the optimal model will be deployed as an online web calculator to explore its feasibility for individualized risk stratification, thereby providing new evidence-based support and practical tools for the precision management of patients with HF.

## 2 Materials and methods

### 2.1 Study design and population

This study is a single-center retrospective cohort study, with data extracted from the electronic medical record system of Beijing Anzhen Hospital, Capital Medical University. The study consecutively included patients hospitalized for heart failure at our institution from January 1, 2024, to June 30, 2025. Inclusion criteria were: (a) first primary diagnosis of heart failure according to the Chinese Guidelines for the Diagnosis and Treatment of Heart Failure 2024; (b) age ≥ 18 years; and (c) complete baseline clinical data upon initial admission. Exclusion criteria were: (a) active malignancy; (b) severe hepatic or renal dysfunction, severe chronic obstructive pulmonary disease with respiratory failure, severe anemia, or active hepatobiliary disease; (c) congenital heart disease or mechanical complications such as ventricular septal perforation or ventricular aneurysm; (d) severe infection or active phase of severe autoimmune disease; and (e) major surgery or severe trauma within 3 months before enrollment. All patients were followed up until December 31, 2025, to ensure a complete 180-day follow-up window. Of the 2,956 enrolled patients, 219 were lost to follow-up, and 2,737 completed the full 180-day follow-up. The primary outcome was the composite of all-cause death or rehospitalization for heart failure within 180 days. Heart failure was diagnosed according to the Chinese Guidelines for the Diagnosis and Treatment of Heart Failure 2024, based on the presence of typical symptoms and/or signs, elevated natriuretic peptide levels, and evidence of structural or functional cardiac abnormalities on echocardiography. Patients were classified by left ventricular ejection fraction (LVEF) into two groups: heart failure with reduced ejection fraction (HFrEF), defined as LVEF ≤ 40%, and heart failure with preserved ejection fraction (HFpEF), defined as LVEF ≥ 50%. Patients with mildly reduced ejection fraction (LVEF 41–49%) were not included in this study.

**Figure 1:**
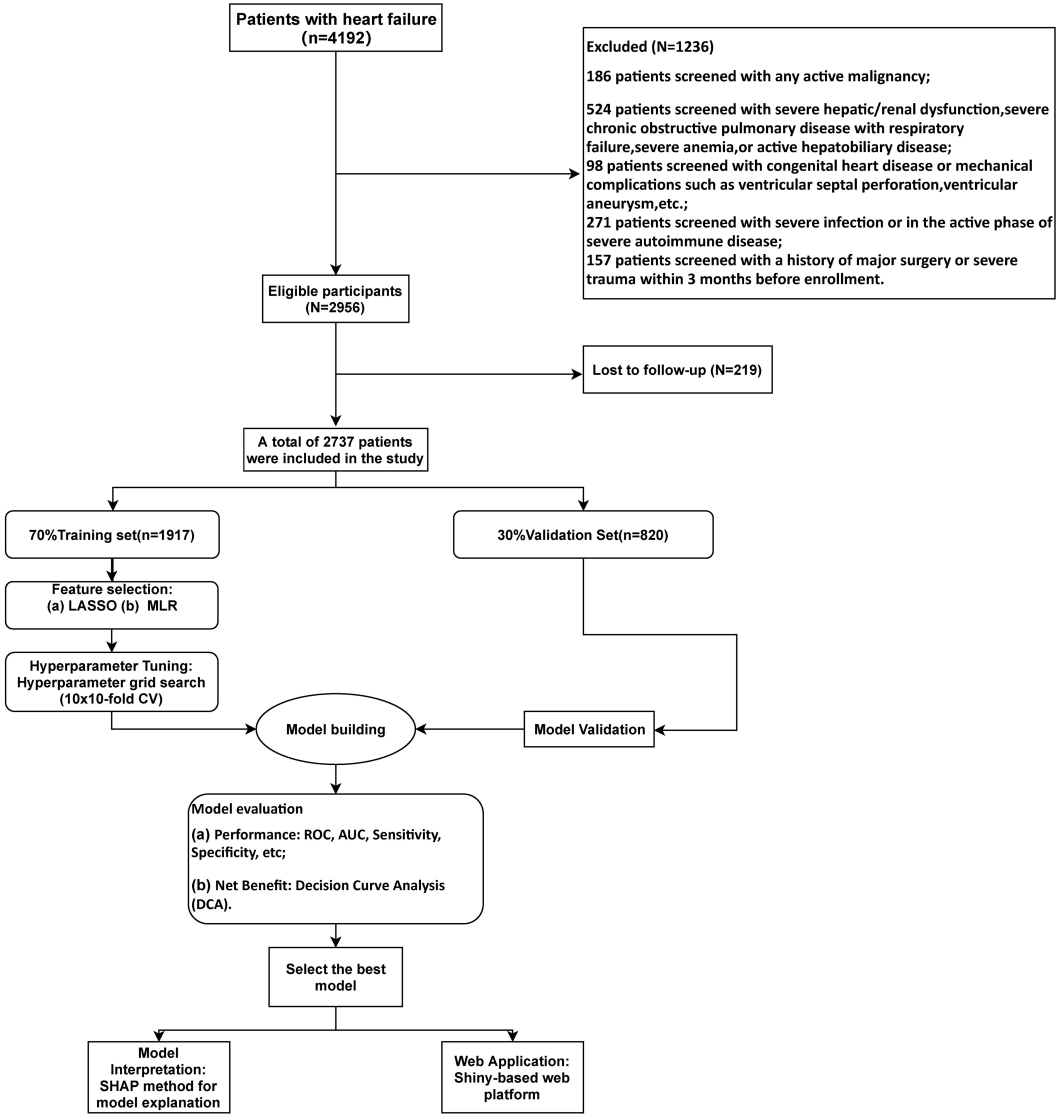
Flowchart of study participant selection, cohort composition, and model development.

Outcome information was primarily obtained through outpatient follow-up records and the medical record query system; for cases with incomplete medical records, telephone follow-up was used as a supplement. The study protocol was approved by our Institutional Ethics Review Board (2026099x), with a waiver of informed consent, and was conducted in accordance with the revised Declaration of Helsinki. The enrollment process is summarized in Figure 1.

### 2.2 Definition of causes of heart failure

Heart failure etiology was classified according to diagnostic codes from the electronic medical records as follows:

- Ischaemic cardiomyopathy (ICM): HF with definitive evidence of coronary artery disease, including a history of myocardial infarction, coronary revascularisation, or ≥50% stenosis in a major epicardial artery on coronary angiography;
- Valvular heart disease (VHD): HF attributed to moderate or severe native valve disease confirmed by echocardiography;
- Dilated cardiomyopathy (DCM): Non-ischaemic HF with left ventricular dilatation and reduced systolic function, after excluding coronary artery disease, valvular disease, and hypertension as aetiologies;
- Hypertensive heart disease (HHD): HF in the setting of long-standing, poorly controlled hypertension, without significant coronary or valvular disease;
- Other: Including congenital heart disease, alcoholic cardiomyopathy, hypertrophic cardiomyopathy, and other rare aetiologies not covered by the above categories.

### 2.3 Variable definition and data preprocessing

To comprehensively assess risk, data were collected at multiple levels: in addition to basic information, the medical history of chronic diseases and incorporated into the basic laboratory testing criteria. History of hyperlipidemia was defined as a clinical diagnosis of hyperlipidemia documented in the electronic medical record at or prior to the index hospitalization for study enrollment. This definition was based on a clear diagnosis recorded by physicians, representing a clinically confirmed state of metabolic abnormality and cumulative atherosclerotic burden. During the data preprocessing stage, variables with a missing rate greater than 10% in the dataset were deleted. For variables with a missing rate less than or equal to 10%, multiple imputation was performed using the mice package in R software, with the imputation method chosen as the random forest algorithm to generate a complete analysis dataset. The data missingness is detailed in Supplementary Table S1.

### 2.4 Laboratory measurements and calculation of UHR

All laboratory data were obtained from the first fasting venous blood sample collected upon hospital admission, ensuring that measurements reflected baseline physiological status prior to inpatient interventions such as diuresis or lipid-lowering therapy. Serum uric acid (UA) and high-density lipoprotein cholesterol (HDL-C) concentrations were measured on a Beckman Coulter AU5831 automated chemistry analyzer (Beckman Coulter, Brea, CA, United States). The Clinical Laboratory of Beijing Anzhen Hospital maintains daily internal quality control using commercial control materials and participates in the external quality assessment program organized by the National Center for Clinical Laboratories (NCCL) of China; intra- and inter-assay coefficients of variation for both analytes remained below 5% throughout the study period. The uric acid-to-high-density lipoprotein cholesterol ratio (UHR) was calculated as: UHR = [UA (μmol/L) / 59.5] / [HDL-C (mmol/L) × 38.67], yielding a dimensionless value.

### 2.5 Outcome definition

The primary outcome of this study was a composite endpoint of all-cause death or rehospitalization due to HF. For brevity and to distinguish from traditional ischemic major adverse cardiovascular events, we refer to this endpoint as the composite outcome throughout the manuscript.

The follow-up period ended at the first occurrence of the composite outcome. Patients were divided into the composite outcome group and the non-composite outcome group based on whether the composite outcome occurred during the follow-up period. Survival time was defined as the number of days from the discharge date to the first occurrence of the composite outcome or the follow-up cutoff date (180 days). Outcome information was obtained through outpatient follow-up visits and medical record reviews.

### 2.6 Statistical analysis

Statistical analysis was performed using R software (version 4.5.1). Continuous variables were tested for normality. Variables with a normal distribution were presented as mean ± standard deviation, and comparisons between groups were performed using the independent samples *t*-test. Variables with a non-normal distribution were presented as median (interquartile range), and comparisons between groups were performed using the Mann–Whitney U test. Categorical variables were presented as frequency (percentage), and comparisons between groups were performed using the chi-square test.

The diagnostic performance of UHR and other composite lipid indices -atherogenic index of plasma (AIP), non-high-density lipoprotein cholesterol to high-density lipoprotein cholesterol ratio (NHHR), and TyG-for the 180-day composite outcome in patients with heart failure was assessed using the ROC curve. A multivariate Cox proportional hazards model was used to evaluate the association between UHR and the risk of the composite outcome. Subgroup analyses (stratified by patient basic informations) were conducted to assess the robustness of the association between UHR and the composite outcome, and interaction was assessed using the likelihood ratio test. All analyses were two-tailed, and a *p*-value < 0.05 was considered statistically significant. The DeLong test was used to compare differences in AUC between different indicators, with UHR compared against AIP, NHHR, and TyG, respectively. Within each subgroup (overall, HFrEF, HFpEF), because three pairwise comparisons were performed, the Bonferroni method was applied to adjust the significance level (adjusted test level α′ = 0.05/3 = 0.0167).

### 2.7 Sensitivity analysis for unmeasured confounding

Given that medication information during hospitalization and prior to admission (e.g., statins, urate-lowering drugs, glucose-lowering drugs, which may directly alter UHR, GA, and sdLDL-C levels) could not be systematically obtained from the retrospective data, a sensitivity analysis using the E-value method was conducted to quantify the potential confounding effect of such factors on the association between UHR and the composite outcome. The E-value represents the minimum strength of association (on a hazard ratio scale) that an unmeasured confounder would need to have with both the exposure (UHR) and the composite outcome, after adjustment for measured covariates, to fully explain away the observed association. E-values were calculated using the EValue package in R, and both the point estimate and the value corresponding to the lower limit of the 95% confidence interval were reported.

### 2.8 Machine learning modeling workflow

Based on six core predictors-body mass index (BMI), history of hyperlipidemia, homocysteine (Hcy), glycated albumin (GA), small dense low-density lipoprotein cholesterol (sdLDL-C), and UHR -jointly selected through prior multivariable logistic regression and least absolute shrinkage and selection operator (LASSO) regression, this study further utilized ML algorithms to construct prediction models for the 180-day composite outcome risk in patients with HF. All modeling work was completed in the Python 3.12 environment.

First, the training set (*n* = 1,917) was used as the model development dataset, and seven classification algorithms were built separately. Parameter tuning was then performed to achieve optimal performance for each model.

Subsequently, the tuned model was finally evaluated on the internal validation set (*n* = 820). By comparing the performance of each model in the validation set, the eXtreme Gradient Boosting (XGBoost) model had the best comprehensive performance in terms of discrimination, calibration, and clinical net benefit; therefore, it was chosen as the final prediction model.

The SHAP framework was used to explain the prediction results of the XGBoost model. Finally, the trained XGBoost model was encapsulated and deployed as an online web calculator to facilitate real-time assessment of the composite outcome risk in patients with HF by clinicians, thereby improving the accessibility and usability of the tool.

## 3 Results

### 3.1 Baseline characteristics of the study population

A total of 2,737 patients with HF were included in this study, of whom 926 (33.8%) experienced the composite outcome during the follow-up period, and 1,811 (66.2%) did not experience the composite outcome. The median age was 62.0 years, and 71.8% of patients were male. Compared with the group without the composite outcome, patients in the composite outcome group had a higher BMI (25.50 vs. 24.80 kg/m^2^, *p* < 0.001) and significantly higher prevalences of hypertension (54.64% vs. 46.00%), diabetes (40.60% vs. 32.74%), and hyperlipidemia (62.63% vs. 48.32%) (all *p* < 0.001). With respect to etiology distribution, ischaemic cardiomyopathy (ICM) was the leading etiology (43.04%), followed by valvular heart disease (VHD, 21.48%), dilated cardiomyopathy (DCM, 17.94%), hypertensive heart disease (HHD, 15.02%), and other aetiologies (2.52%). The proportion of ICM was significantly higher in the composite outcome group than in the group without the composite outcome (51.46% vs. 38.73%, *p* < 0.001), whereas the proportions of VHD and HHD were higher in the group without the composite outcome (both *p* < 0.05). The distribution of DCM showed no significant difference between the two groups (*p* = 0.283).

In terms of laboratory indicators, baseline brain natriuretic peptide (BNP) did not differ significantly between the composite outcome group and the group without the composite outcome (BNP: median 146.00 vs. 135.00 pg./mL, *p* = 0.426). In addition to BNP, this study further compared laboratory indicators reflecting different dimensions of heart failure severity between the two groups. There were no statistically significant differences between the composite outcome group and the group without the composite outcome in estimated glomerular filtration rate (eGFR: 80.81 vs. 80.18 mL/min/1.73 m^2^, *p* = 0.540), urea (Urea: 6.72 vs. 6.72 mmol/L, *p* = 0.353), and high-sensitivity C-reactive protein (hs-CRP: 2.90 vs. 3.39 mg/L, *p* = 0.597). Among the nutrition-related indicators, the absolute between-group differences in medians were 0.40 g/L for serum albumin (Alb: 41.80 vs. 41.40 g/L, *p* = 0.024) and 0.01 g/L for prealbumin (PA: 0.20 vs. 0.19 g/L, *p* = 0.024), respectively. Furthermore, patients in the composite outcome group had significantly higher levels of uric acid (UA), homocysteine (Hcy), small dense low-density lipoprotein cholesterol (sdLDL-C), cystatin C (CysC), atherogenic index of plasma (AIP), uric acid-to-high-density lipoprotein cholesterol ratio (UHR), and non-high-density lipoprotein cholesterol to high-density lipoprotein cholesterol ratio (NHHR) than the group without the composite outcome. Other indicators such as creatinine (Cr), aspartate aminotransferase (AST), and total bile acid (TBA) also showed statistical differences between the two groups (*p* < 0.05). Detailed baseline characteristics of the overall population are presented in Table 1.

**Table 1:** Baseline characteristics of the overall study population.

Variables	Total (*n* = 2,737)	No composite outcome (*n* = 1,811)	Composite outcome (*n* = 926)	*p*
HFpEF (%)	1,816 (66.35)	1,175 (64.88)	641 (69.22)	
HFrEF (%)	921 (33.65)	636 (35.12)	285 (30.78)	
LVEF (median [IQR])	59.00 [36.00, 64.00]	58.00 [35.00, 64.00]	60.00 [37.00, 65.00]	0.026
Causes of heart failure, *n* (%)				
Ischaemic cardiomyopathy (%)	1,178 (43.04)	701 (38.73)	477 (51.46)	<0.001
Valvular heart disease (%)	588 (21.48)	440 (24.31)	148 (15.97)	<0.001
Dilated cardiomyopathy (%)	491 (17.94)	314 (17.35)	177 (19.09)	0.283
Hypertensive heart disease (%)	411 (15.02)	291 (16.08)	120 (12.94)	0.034
Other (%)	69 (2.52)	54 (2.98)	15 (1.62)	0.043
Age (median [IQR])	62.00 [53.00, 70.00]	62.00 [53.00, 70.00]	62.00 [54.00, 70.00]	0.579
BMI (median [IQR])	25.10 [22.90, 27.20]	24.80 [22.50, 27.00]	25.50 [23.50, 27.70]	<0.001
Sex (%)				0.119
Female	771 (28.17)	528 (29.16)	243 (26.24)	
Male	1,966 (71.83)	1,283 (70.84)	683 (73.76)	
Hypertension (%)	1,339 (48.92)	833 (46.00)	506 (54.64)	<0.001
Diabetes (%)	969 (35.40)	593 (32.74)	376 (40.60)	<0.001
Hyperlipidemia (%)	1,455 (53.16)	875 (48.32)	580 (62.63)	<0.001
Cr (median [IQR]), (μmol/L)	79.50 [64.80, 98.90]	78.90 [63.45, 98.30]	80.85 [66.93, 99.50]	0.027
PA (median [IQR]), (g/L)	0.20 [0.10, 0.25]	0.19 [0.09, 0.25]	0.20 [0.11, 0.25]	0.024
Hcy (median [IQR]), (μmol/L)	12.03 [7.42, 16.07]	11.54 [6.74, 15.52]	12.88 [8.53, 16.89]	<0.001
hs-CRP (median [IQR]), (mg/L)	3.15 [0.71, 20.00]	3.39 [0.69, 20.00]	2.90 [0.75, 20.00]	0.597
ALT (median [IQR]), (U/L)	18.00 [12.00, 29.00]	18.00 [12.00, 29.00]	19.00 [13.00, 28.00]	0.269
AST (median [IQR]), (U/L)	19.86 [15.36, 26.88]	19.20 [15.26, 26.64]	20.80 [16.64, 27.04]	<0.001
Alb (median [IQR]), (g/L)	41.50 [37.10, 44.90]	41.40 [36.80, 44.80]	41.80 [37.70, 45.18]	0.024
TB (median [IQR]), (μmol/L)	12.70 [8.70, 18.61]	12.92 [8.80, 18.88]	12.30 [8.49, 18.16]	0.213
DB (median [IQR]), (μmol/L)	4.00 [2.58, 6.11]	4.00 [2.58, 6.16]	3.95 [2.55, 6.03]	0.803
ALP (median [IQR]), (U/L)	78.00 [56.00, 117.00]	78.00 [56.00, 120.50]	79.00 [58.12, 114.88]	0.766
GGT (median [IQR]), (U/L)	37.44 [16.64, 240.96]	37.44 [16.32, 240.96]	39.52 [18.72, 247.52]	0.045
TBA (median [IQR]), (μmol/L)	4.00 [1.80, 6.70]	3.80 [1.80, 6.40]	4.20 [1.88, 7.07]	0.018
ChE (median [IQR]), (kU/L)	6.40 [3.80, 8.30]	6.40 [3.60, 8.20]	6.40 [3.82, 8.30]	0.738
Urea (median [IQR]), (mmol/L)	6.72 [5.18, 9.05]	6.72 [5.16, 8.90]	6.72 [5.21, 9.30]	0.353
UA (median [IQR]), (μmol/L)	323.86 [248.54, 411.29]	309.42 [231.85, 393.25]	358.63 [282.92, 445.52]	<0.001
Glu (median [IQR]), (mmol/L)	5.65 [4.80, 7.17]	5.60 [4.78, 7.04]	5.73 [4.89, 7.26]	0.053
CK (median [IQR]), (U/L)	64.00 [40.00, 104.10]	65.00 [40.00, 107.00]	63.00 [39.00, 100.07]	0.198
LD (median [IQR]), (U/L)	182.00 [149.00, 228.00]	183.00 [149.00, 229.00]	182.00 [149.05, 225.75]	0.738
TG (median [IQR]), (mmol/L)	1.34 [0.82, 2.15]	1.31 [0.83, 2.09]	1.36 [0.82, 2.18]	0.29
TC (median [IQR]), (mmol/L)	3.38 [2.29, 4.26]	3.46 [2.31, 4.41]	3.26 [2.26, 4.07]	0.001
HDL-C (median [IQR]), (mmol/L)	1.00 [0.69, 1.23]	1.03 [0.71, 1.28]	0.92 [0.68, 1.12]	<0.001
LDL-C (median [IQR]), (mmol/L)	1.79 [0.90, 2.58]	1.82 [0.89, 2.64]	1.75 [0.90, 2.44]	0.062
Ca (median [IQR]), (mmol/L)	2.29 [2.07, 2.41]	2.29 [2.06, 2.41]	2.30 [2.09, 2.41]	0.635
GA (median [IQR]), (g/dl)	13.27 [9.55, 15.48]	12.90 [8.74, 14.88]	14.12 [10.88, 16.35]	<0.001
eGFR (median [IQR]), (mL/min/1.73 m^2^)	80.47 [56.30, 95.82]	80.18 [56.47, 96.11]	80.81 [56.27, 94.97]	0.54
LPa (median [IQR]), nmol/L	23.30 [8.20, 65.00]	23.30 [7.90, 62.85]	24.40 [8.40, 68.80]	0.334
sdLDL-C (median [IQR]), mmol/L	0.18 [0.06, 0.55]	0.17 [0.06, 0.51]	0.22 [0.06, 0.61]	<0.001
CysC (median [IQR]), mg/L	1.15 [0.56, 1.54]	1.11 [0.53, 1.49]	1.21 [0.85, 1.65]	<0.001
BNP (median [IQR]), pg./ml	138.00 [46.00, 487.00]	135.00 [47.00, 446.00]	146.00 [42.00, 550.00]	0.426
AIP (median [IQR])	1.20 [0.74, 1.71]	1.15 [0.70, 1.66]	1.30 [0.83, 1.81]	<0.001
UHR (median [IQR])	0.15 [0.10, 0.23]	0.13 [0.09, 0.20]	0.17 [0.12, 0.27]	<0.001
NHHR (median [IQR])	2.42 [1.74, 3.38]	2.36 [1.70, 3.28]	2.53 [1.82, 3.56]	0.001
TyG (median [IQR])	4.69 [4.42, 4.98]	4.67 [4.42, 4.97]	4.72 [4.42, 5.01]	0.062

IQR, interquartile range; HFrEF, Heart Failure with reduced Ejection Fraction; HFpEF, Heart Failure with preserved Ejection Fraction; BMI, body mass index; Cr, creatinine; PA, prealbumin; Hcy, homocysteine; hs-CRP, high-sensitivity C-reactive protein; ALT, alanine aminotransferase; AST, aspartate aminotransferase; Alb, albumin; TB, total bilirubin; DB, direct bilirubin; ALP, alkaline phosphatase; GGT, gamma-glutamyl transferase; TBA, total bile acid; ChE, cholinesterase; UA, uric acid; Glu, glucose; CK, creatine kinase; LD, lactate dehydrogenase; TG, triglycerides; TC, total cholesterol; HDL-C, high-density lipoprotein cholesterol; LDL-C, low-density lipoprotein cholesterol; Ca, calcium; GA, glycoalbumin; eGFR, estimated glomerular filtration rate; LPa, lipoprotein(a); sdLDL-C, small dense low-density lipoprotein cholesterol; CysC, cystatin C; BNP, brain natriuretic peptide; AIP, atherogenic index of plasma; UHR, uric acid to high-density lipoprotein cholesterol ratio; NHHR, non-high-density lipoprotein cholesterol to high-density lipoprotein cholesterol ratio; TyG, triglyceride-glucose index; LVEF, Left Ventricular Ejection Fraction.

To construct the prediction model, the overall population was randomly divided into a training set (*n* = 1,917) and an internal validation set (*n* = 820) in a 7:3 ratio. The incidence rate of the composite outcome was 33.9% in the training set and 33.8% in the validation set, consistent with the overall population. The distribution of key variables and the trends of differences between groups in the training and validation sets were highly similar to those in the overall population, indicating that the random grouping was reasonable and the data partitioning was balanced. Detailed baseline characteristics of the training set and internal validation set are shown in Supplementary Tables S2, S3, respectively. The baseline characteristics of the training and validation sets were well balanced (Table 2), and the incidence of the composite outcome was nearly identical between the two sets (33.9% vs. 33.8%).

**Table 2:** Comparison of baseline characteristics between the training and validation sets.

Variables	Total (*n* = 2,737)	Training set (*n* = 1,917)	Validation set (*n* = 820)	*p*
HFpEF (%)	1,816 (66.35)	1,286 (67.08)	530 (64.63)	
HFrEF (%)	921 (33.65)	631 (32.92)	290 (35.37)	
LVEF (median [IQR])	59.00 [36.00, 64.00]	59.00 [36.00, 64.00]	58.00 [35.00, 64.00]	0.284
Causes of heart failure, *n* (%)				
Ischaemic cardiomyopathy (%)	1,178 (43.04)	842 (43.95)	336 (40.93)	0.156
Valvular heart disease (%)	588 (21.48)	408 (21.29)	180 (21.92)	0.751
Dilated cardiomyopathy (%)	491 (17.94)	347 (18.11)	144 (17.54)	0.762
Hypertensive heart disease (%)	411 (15.02)	279 (14.56)	132 (16.08)	0.337
Other (%)	69 (2.52)	45 (2.35)	24 (2.92)	0.456
Age (median [IQR])	62.00 [53.00, 70.00]	62.00 [53.00, 70.00]	61.00 [52.00, 70.00]	0.146
BMI (median [IQR])	25.10 [22.90, 27.20]	25.10 [22.80, 27.20]	25.00 [22.90, 27.20]	<0.001
Sex (%)				0.067
Female	771 (28.17)	535 (27.91)	236 (28.78)	
Male	1,966 (71.83)	1,382 (72.09)	584 (71.22)	
Hypertension (%)	1,339 (48.92)	935 (48.77)	404 (49.27)	0.088
Diabetes (%)	969 (35.40)	677 (35.32)	292 (35.61)	0.012
Hyperlipidemia (%)	1,455 (53.16)	1,036 (54.04)	419 (51.10)	<0.001
Cr (median [IQR]), (μmol/L)	79.50 [64.80, 98.90]	79.20 [64.80, 98.90]	80.65 [64.40, 98.82]	0.051
PA (median [IQR]), (g/L)	0.20 [0.10, 0.25]	0.20 [0.10, 0.25]	0.19 [0.11, 0.25]	0.101
Hcy (median [IQR]), (μmol/L)	12.03 [7.42, 16.07]	11.93 [7.08, 16.00]	12.14 [7.94, 16.20]	<0.001
hs-CRP (median [IQR]), (mg/L)	3.15 [0.71, 20.00]	2.96 [0.67, 20.00]	3.80 [0.79, 20.25]	0.48
ALT (median [IQR]), (U/L)	18.00 [12.00, 29.00]	18.00 [12.00, 29.00]	19.00 [13.00, 29.00]	0.537
AST (median [IQR]), (U/L)	19.86 [15.36, 26.88]	19.76 [15.36, 25.92]	20.16 [15.36, 28.80]	0.028
Alb (median [IQR]), (g/L)	41.50 [37.10, 44.90]	41.50 [37.10, 44.90]	41.40 [37.10, 44.90]	0.008
TB (median [IQR]), (μmol/L)	12.70 [8.70, 18.61]	12.60 [8.57, 18.30]	13.10 [9.05, 19.60]	0.398
DB (median [IQR]), (μmol/L)	4.00 [2.58, 6.11]	3.92 [2.50, 5.99]	4.10 [2.70, 6.47]	0.826
ALP (median [IQR]), (U/L)	78.00 [56.00, 117.00]	78.00 [56.00, 115.00]	79.00 [57.00, 122.25]	0.876
GGT (median [IQR]), (U/L)	37.44 [16.64, 240.96]	37.44 [16.54, 240.96]	39.68 [17.28, 240.96]	0.069
TBA (median [IQR]), (μmol/L)	4.00 [1.80, 6.70]	3.90 [1.80, 6.60]	4.00 [1.80, 6.73]	0.04
ChE (median [IQR]), (kU/L)	6.40 [3.80, 8.30]	6.30 [3.50, 8.20]	6.40 [3.90, 8.40]	0.612
Urea (median [IQR]), (mmol/L)	6.72 [5.18, 9.05]	6.76 [5.20, 9.03]	6.63 [5.17, 9.08]	0.514
UA (median [IQR]), (μmol/L)	323.86 [248.54, 411.29]	324.33 [248.62, 411.06]	321.23 [248.21, 412.58]	<0.001
Glu (median [IQR]), (mmol/L)	5.65 [4.80, 7.17]	5.64 [4.77, 7.14]	5.69 [4.87, 7.23]	0.036
CK (median [IQR]), (U/L)	64.00 [40.00, 104.10]	65.00 [40.00, 105.00]	63.00 [39.00, 104.10]	0.286
LD (median [IQR]), (U/L)	182.00 [149.00, 228.00]	181.00 [148.00, 227.00]	186.00 [152.00, 230.00]	0.679
TG (median [IQR]), (mmol/L)	1.34 [0.82, 2.15]	1.32 [0.82, 2.14]	1.35 [0.84, 2.15]	0.588
TC (median [IQR]), (mmol/L)	3.38 [2.29, 4.26]	3.38 [2.23, 4.25]	3.39 [2.39, 4.30]	0.001
HDL-C (median [IQR]), (mmol/L)	1.00 [0.69, 1.23]	0.99 [0.68, 1.22]	1.02 [0.72, 1.24]	<0.001
LDL-C (median [IQR]), (mmol/L)	1.79 [0.90, 2.58]	1.81 [0.86, 2.55]	1.78 [0.95, 2.63]	0.034
Ca (median [IQR]), (mmol/L)	2.29 [2.07, 2.41]	2.29 [2.07, 2.41]	2.30 [2.09, 2.41]	0.839
GA (median [IQR]), (g/dl)	13.27 [9.55, 15.48]	13.25 [8.79, 15.49]	13.35 [9.95, 15.38]	<0.001
eGFR (median [IQR]), (mL/min/1.73 m^2^)	80.47 [56.30, 95.82]	79.95 [56.09, 95.78]	81.10 [57.48, 96.12]	0.774
LPa (median [IQR]), nmol/L	23.30 [8.20, 65.00]	22.20 [7.90, 63.90]	24.15 [8.57, 67.17]	0.495
sdLDL-C (median [IQR]), mmol/L	0.18 [0.06, 0.55]	0.18 [0.06, 0.56]	0.19 [0.06, 0.50]	0.003
CysC (median [IQR]), mg/L	1.15 [0.56, 1.54]	1.15 [0.51, 1.53]	1.17 [0.79, 1.55]	<0.001
BNP (median [IQR]), pg./ml	138.00 [46.00, 487.00]	130.00 [44.00, 481.00]	158.50 [49.00, 499.25]	0.364
AIP (median [IQR])	1.20 [0.74, 1.71]	1.21 [0.73, 1.71]	1.17 [0.74, 1.71]	<0.001
UHR (median [IQR])	0.15 [0.10, 0.23]	0.15 [0.10, 0.23]	0.14 [0.10, 0.23]	<0.001
NHHR (median [IQR])	2.42 [1.74, 3.38]	2.41 [1.74, 3.38]	2.43 [1.75, 3.41]	0.02
TyG (median [IQR])	4.69 [4.42, 4.98]	4.68 [4.40, 4.98]	4.70 [4.44, 4.99]	0.169

IQR, interquartile range; HFrEF, Heart Failure with reduced Ejection Fraction; HFpEF, Heart Failure with preserved Ejection Fraction; BMI, body mass index; Cr, creatinine; PA, prealbumin; Hcy, homocysteine; hs-CRP, high-sensitivity C-reactive protein; ALT, alanine aminotransferase; AST, aspartate aminotransferase; Alb, albumin; TB, total bilirubin; DB, direct bilirubin; ALP, alkaline phosphatase; GGT, gamma-glutamyl transferase; TBA, total bile acid; ChE, cholinesterase; UA, uric acid; Glu, glucose; CK, creatine kinase; LD, lactate dehydrogenase; TG, triglycerides; TC, total cholesterol; HDL-C, high-density lipoprotein cholesterol; LDL-C, low-density lipoprotein cholesterol; Ca, calcium; GA, glycoalbumin; eGFR, estimated glomerular filtration rate; LPa, lipoprotein(a); sdLDL-C, small dense low-density lipoprotein cholesterol; CysC, cystatin C; BNP, brain natriuretic peptide; AIP, atherogenic index of plasma; UHR, uric acid to high-density lipoprotein cholesterol ratio; NHHR, non-high-density lipoprotein cholesterol to high-density lipoprotein cholesterol ratio; TyG, triglyceride-glucose index; LVEF, Left Ventricular Ejection Fraction.

### 3.2 Predictive efficacy of UHR for the composite outcome in patients with heart failure

**Figure 2:**
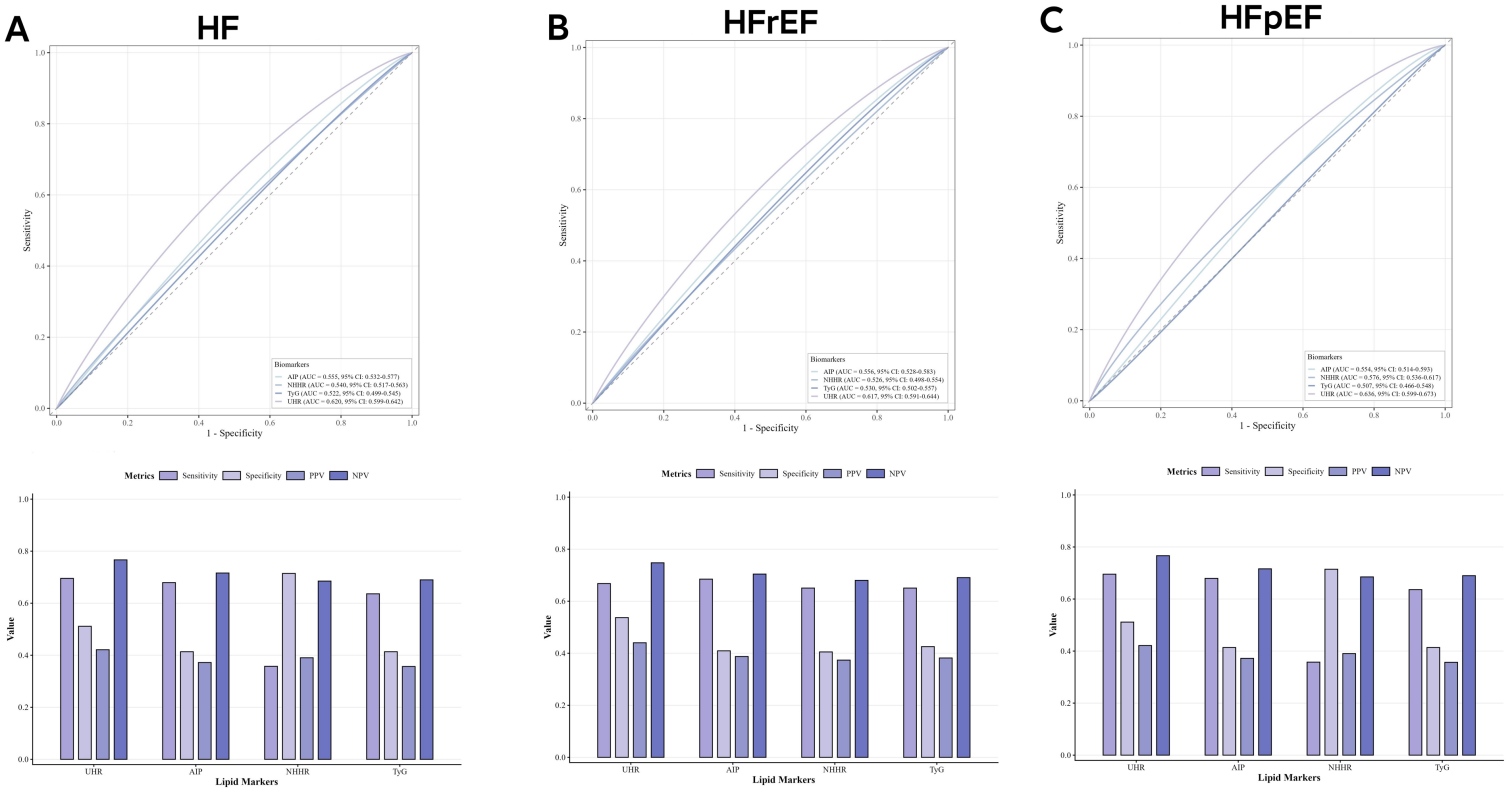
ROC curves of UHR and other composite indices for predicting the composite outcome in heart failure patients. **(A)** Overall heart failure patients; **(B)** Heart failure with reduced ejection fraction (HFrEF); **(C)** Heart failure with preserved ejection fraction (HFpEF). UHR, uric acid-to-high-density lipoprotein cholesterol ratio; ROC, receiver operating characteristic curve; AUC, area under the curve; HF, heart failure; HFrEF, heart failure with reduced ejection fraction; HFpEF, heart failure with preserved ejection fraction.

The receiver operating characteristic (ROC) curve was used to evaluate the predictive value of the UHR for the composite outcome within 180 days in patients with heart failure (Figure 2). The AUC values and DeLong test results for each indicator are detailed in Supplementary Table S4.

In the overall heart failure population, the AUC of UHR was 0.620 (95% CI, 0.599–0.642), which was significantly higher than that of AIP (AUC = 0.555), NHHR (AUC = 0.540), and TyG (AUC = 0.522); all differences remained statistically significant after Bonferroni correction for multiple comparisons (all adjusted *p* < 0.001).

In the heart failure with reduced ejection fraction (HFrEF) subgroup, the AUC of UHR was 0.636 (95% CI, 0.599–0.673), significantly higher than that of AIP (AUC = 0.554, adjusted *p* < 0.001) and TyG (AUC = 0.507, adjusted *p* < 0.001); the difference between UHR and NHHR (AUC = 0.576) was not statistically significant after correction (adjusted *p* = 0.024 > 0.0167).

In the heart failure with preserved ejection fraction (HFpEF) subgroup, the AUC of UHR was 0.617 (95% CI: 0.591–0.644), significantly higher than that of AIP (AUC = 0.556), NHHR (AUC = 0.526), and TyG (AUC = 0.530), with all differences remaining significant after correction (all adjusted *p* < 0.001). These results indicate that UHR has an overall superior predictive performance for the 180-day composite outcome in heart failure patients compared with other composite lipid indices, with the most robust advantage observed in the HFpEF subgroup.

We further compared the predictive performance of UHR with several established heart failure prognostic biomarkers, including BNP, eGFR, CysC, hs-CRP, Cr, Hcy, and creatine kinase. As summarized in Supplementary Table S5, UHR (AUC = 0.620) outperformed all these conventional markers in predicting the 180-day composite outcome.

### 3.3 Relation between UHR and the composite outcome risk in patients with heart failure

UHR was divided into quartiles (Q1–Q4), with the lowest quartile (Q1) as the reference. Multivariable Cox regression was used to assess its relation with the risk of the 180-day composite outcome in patients with HF (Table 3).

**Table 3:** Association between UHR and the risk of the composite outcome in patients with heart failure.

UHR	Model 1		Model 2		Model 3	
	HR (95% CI)	*P*-value	HR (95% CI)	*P*-value	HR (95% CI)	*P*-value
HF						
Q1	Reference		Reference		Reference	
Q2	1.684 (1.353–2.095)	<0.001	1.688 (1.354–2.103)	<0.001	1.695 (1.360–2.113)	<0.001
Q3	2.635 (2.147–3.235)	<0.001	2.600 (2.112–3.202)	<0.001	2.540 (2.062–3.129)	<0.001
Q4	2.574 (2.095–3.162)	<0.001	2.571 (2.086–3.168)	<0.001	2.499 (2.027–3.081)	<0.001
PerSD	1.179 (1.126–1.235)	<0.001	1.178 (1.123–1.236)	<0.001	1.170 (1.113–1.229)	<0.001
HFrEF						
Q1	Reference		Reference		Reference	
Q2	2.774 (1.828–4.210)	<0.001	2.811 (1.849–4.273)	<0.001	2.820 (1.855–4.288)	<0.001
Q3	2.898 (1.913–4.391)	<0.001	2.943 (1.938–4.472)	<0.001	2.947 (1.939–4.478)	<0.001
Q4	3.975 (2.656–5.948)	<0.001	4.035 (2.686–6.061)	<0.001	3.989 (2.653–5.998)	<0.001
PerSD	1.264 (1.161–1.377)	<0.001	1.261 (1.157–1.374)	<0.001	1.242 (1.137–1.357)	<0.001
HFpEF						
Q1	Reference		Reference		Reference	
Q2	1.561 (1.205–2.022)	<0.001	1.540 (1.186–2.000)	0.001	1.533 (1.180–1.993)	0.001
Q3	2.567 (2.018–3.265)	<0.001	2.491 (1.949–3.184)	<0.001	2.393 (1.870–3.062)	<0.001
Q4	2.286 (1.791–2.919)	<0.001	2.251 (1.755–2.886)	<0.001	2.164 (1.686–2.777)	<0.001
PerSD	1.151 (1.088–1.217)	<0.001	1.148 (1.083–1.217)	<0.001	1.137 (1.069–1.208)	<0.001

Data are presented as hazard ratio (95% confidence interval). Model 1: unadjusted; Model 2: adjusted for age, sex, and BMI; Model 3: adjusted for age, sex, BMI, hypertension, diabetes, and hyperlipidemia.

UHR, uric acid-to-high-density lipoprotein cholesterol ratio; CI, confidence interval; HR, hazard ratio; Q1–Q4, quartiles (Q1 as the lowest quartile); PerSD, per standard deviation increase; BMI, body mass index; HFrEF, heart failure with reduced ejection fraction; HFpEF, heart failure with preserved ejection fraction.

In the Model 3, UHR levels were significantly and independently positively associated with the composite outcome risk. In the overall patient population with HF, the risk of the composite outcome in Q4 was 2.499 times that in Q1 (95% CI: 2.027–3.081), and each one-SD increase (PerSD) was associated with a 17.0% increase in risk (95% CI: 1.113–1.229). In the HFrEF subgroup, the relation was even stronger: the HR for Q4 was 3.989 (95% CI: 2.653–5.998), and the PerSD HR was 1.242 (95% CI: 1.137–1.357). In the HFpEF subgroup, the relation was also significant. All of these results had *p* < 0.001. These findings indicate that UHR is an independent risk factor for the composite outcome in patients with HF, and this relation remains stable in both the HFrEF and HFpEF subtypes. To evaluate the impact of unmeasured confounding on the association between UHR and the composite outcome, this study calculated the E-value. For each standard deviation increase in UHR, the hazard ratio (HR) was 1.17 (95% CI: 1.11–1.23); the E-value for the point estimate was 1.62, and the E-value for the lower limit of the 95% confidence interval was 1.47.

### 3.4 Subgroup analysis

**Figure 3:**
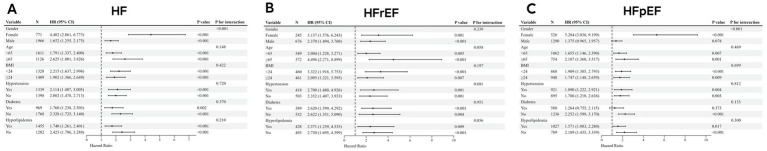
Subgroup analysis of the association between UHR and the composite outcome. Forest plot displaying hazard ratios (HRs) and 95% confidence intervals (CIs) for the composite outcome per unit increase in UHR across different subgroups. **(A)** Overall heart failure patients; **(B)** Heart failure with reduced ejection fraction (HFrEF); **(C)** Heart failure with preserved ejection fraction (HFpEF). All models were adjusted for age, sex, BMI, hypertension, diabetes, and hyperlipidemia (except for the stratifying variable). *p* values for interaction are indicated. UHR, uric acid-to-high-density lipoprotein cholesterol ratio; HR, hazard ratio; CI, confidence interval; HF, heart failure; HFrEF, heart failure with reduced ejection fraction; HFpEF, heart failure with preserved ejection fraction.

To evaluate the robustness of the relation between UHR and the composite outcome, stratified analysis based on patient basic informations (Figure 3). The models were adjusted for patient basic informations (except for the stratifying variable).

In the overall patient population with HF (Figure 3A), the relation between UHR and the composite outcome was significant in all subgroups (*p* < 0.05). Interaction tests showed a significant interaction with sex (P for interaction < 0.001), with a stronger relation in females (HR = 4.402 vs. 1.652).

In the HFrEF subgroup (Figure 3B), the relation between UHR and the composite outcome was significant in all subgroups (*p* < 0.05), and no significant interactions were observed (P for interaction > 0.05).

In the HFpEF subgroup (Figure 3C), the relation between UHR and the composite outcome was significant in most subgroups. Interaction tests showed a significant interaction with sex (P for interaction < 0.001), with a significant relation in females (HR = 5.284) but no significant relation in males or diabetic patients.

These results indicate that the relation between UHR and the composite outcome is robust in the all population, but there is a modifying effect of sex in patients with HFpEF. *Post hoc* sensitivity analysis showed that the male HFpEF subgroup (*n* = 1,290, number of events = 470) had a statistical power of 92.8%, indicating an adequate sample size; the failure to detect a significant association suggests that the true effect in males may be weak. In the female HFpEF subgroup (*n* = 526, number of events = 171), if calculated based on the observed effect size (HR = 5.284), the statistical power approached 100%, but this only reflects the conditional probability assuming the true HR is indeed 5.28. Given that effect sizes are often overestimated in small-sample subgroups, the true HR could be substantially lower than this observed value. If the true HR were assumed to be 1.5 or 1.2, the power of this subgroup would drop to 74.4 and 22%, respectively. Therefore, the conclusion regarding this interaction should be interpreted with caution: the effect size may be inflated due to the limited sample size, and the definitive sex difference awaits validation in larger-scale studies.

### 3.5 Dose–response relationship between UHR and the composite outcome risk

**Figure 4:**
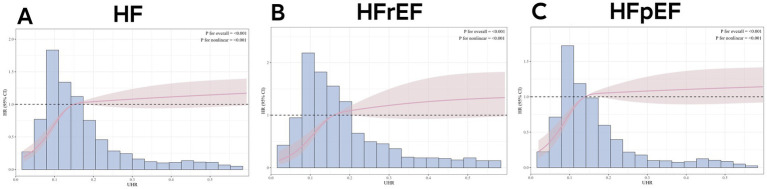
Dose–response relationship between UHR and the composite outcome risk. Restricted cubic spline plots showing the association of UHR with the composite outcome risk. **(A)** Overall heart failure patients; **(B)** Heart failure with reduced ejection fraction (HFrEF); **(C)** Heart failure with preserved ejection fraction (HFpEF). All models were adjusted for age, sex, BMI, hypertension, diabetes, and hyperlipidemia. Solid lines represent hazard ratios (HRs); shaded areas indicate 95% confidence intervals (CIs). Overall *p* < 0.001 and *P* for nonlinear < 0.001 for all models. UHR, uric acid-to-high-density lipoprotein cholesterol ratio; HR, hazard ratio; CI, confidence interval; HF, heart failure; HFrEF, heart failure with reduced ejection fraction; HFpEF, heart failure with preserved ejection fraction.

RCS were used to further explore the dose–response relationship between UHR and the risk of the 180-day composite outcome in patients with HF (Figure 4). The models were adjusted for age, sex, BMI, hypertension, diabetes, and hyperlipidemia. In this study, the restricted cubic spline (RCS) employed 3 knots, with knot positions at the 10th, 50th, and 90th percentiles of the variable distribution (the default setting of the rms package).

The RCS analysis showed a significant nonlinear association between UHR and the composite outcome risk (overall *p* < 0.001, nonlinear *p* < 0.001). Risk increased only modestly at lower UHR levels but rose steeply once UHR exceeded approximately 0.15, suggesting a threshold effect. This pattern was consistent in the overall cohort and in both the HFrEF and HFpEF subgroups (Figure 4). In the HFrEF and HFpEF subgroups, significant nonlinear relationships were also observed (all P for nonlinear < 0.001), suggesting a threshold effect of UHR on the composite outcome risk (Figures 4B,C).

### 3.6 Kaplan–Meier survival analysis

**Figure 5:**
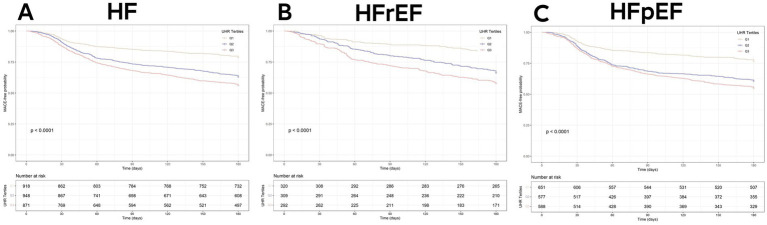
Kaplan–Meier curves for composite outcome-free probability in heart failure patients stratified by UHR tertiles. **(A)** Overall heart failure patients; **(B)** Heart failure with reduced ejection fraction (HFrEF); **(C)** Heart failure with preserved ejection fraction (HFpEF). UHR was divided into tertiles (Q1–Q3). Log-rank *p* < 0.001 for all comparisons. UHR, uric acid-to-high-density lipoprotein cholesterol ratio; HF, heart failure; HFrEF, heart failure with reduced ejection fraction; HFpEF, heart failure with preserved ejection fraction.

Kaplan–Meier curves were used to compare the cumulative probability of remaining free of the composite outcome within 180 days among patients with HF stratified by different levels of UHR (tertiles) (Figure 5). The log-rank test showed that, regardless of whether it was for the overall patient population with HF (Figure 5A), the HFrEF subgroup (Figure 5B), or the HFpEF subgroup (Figure 5C), the differences in survival distribution among the three groups were statistically significant (all *p* < 0.001). The higher the UHR, the lower the probability of remaining free of the composite outcome: Q3 < Q2 < Q1. These results indicate that increasing UHR is closely related with an increased risk of the composite outcome.

### 3.7 Sensitivity analysis by joint stratification of UA and HDL-C

**Figure 6:**
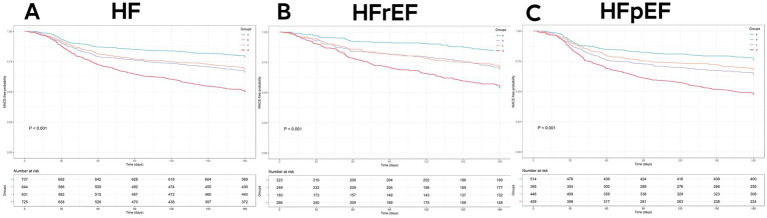
Kaplan–Meier survival curves stratified by combined UA and HDL-C categories for the composite outcome. Patients were classified into four groups based on median values of UA and HDL-C: a = low UA + high HDL-C (reference); b = high UA + high HDL-C (isolated high UA); c = low UA + low HDL-C (isolated low HDL-C); d = high UA + low HDL-C (combined risk). **(A)** All HF patients; **(B)** HFrEF subgroup; **(C)** HFpEF subgroup. *p* values were calculated by log-rank test, with three decimal places shown.

To clarify whether the prognostic value of UHR is driven by elevated uric acid, decreased HDL-C, or their synergistic effect, patients were further divided into four groups based on the medians of UA and HDL-C, and Kaplan–Meier survival curves were plotted (Figure 6).

In the overall heart failure population (Figure 6A), the 180-day probability of remaining free of the composite outcome differed significantly among the four groups (log-rank *p* < 0.001), ranked from highest to lowest as follows: group a (low UA + high HDL-C, reference) > group c (low UA + low HDL-C) > group b (high UA + high HDL-C) > group d (high UA + low HDL-C, combined risk). Group d had the lowest probability of remaining free of the composite outcome and group a the highest, indicating that patients with both high uric acid and low HDL-C had the worst prognosis.

In the HFrEF subgroup (Figure 6B), the differences among the four groups were also significant (log-rank p < 0.001), with the ranking a > b ≈ c > d. The curves for group b (isolated high UA) and group c (isolated low HDL-C) nearly overlapped, suggesting that in HFrEF patients, isolated UA elevation and isolated HDL-C reduction confer similar increases in risk, while the coexistence of both further escalates the risk.

In the HFpEF subgroup (Figure 6C), the four groups were ranked as a > c > b > d (log-rank *p* < 0.001). The probability of remaining free of the composite outcome was higher in group c than in group b, implying that in HFpEF patients, the isolated effect of low HDL-C may be smaller than that of isolated high UA; however, when both conditions coexist (group d), the risk remains the highest.

### 3.8 Feature selection

**Figure 7:**
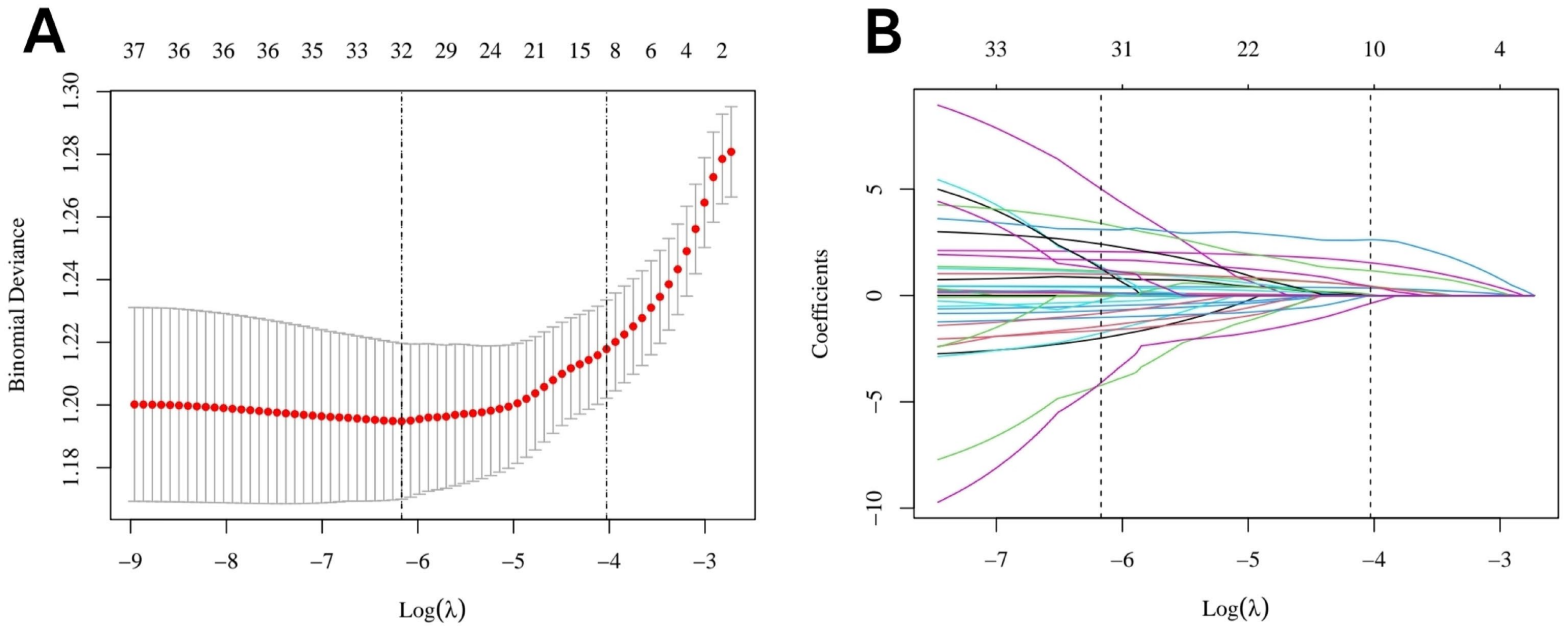
Feature selection process using LASSO regression. **(A)** LASSO coefficient path plot. **(B)** 10-fold cross-validation error curve.

To screen for core predictors, variables with *p* < 0.05 in the univariate analysis were first included in multivariable logistic regression, which identified 11 independently associated factors (*p* < 0.05). Simultaneously, LASSO regression was used for penalized feature selection. The optimal penalty parameter *λ* was determined by 10-fold cross-validation, using the value corresponding to one standard error of the minimum criterion (λ = 0.01778839), yielding 10 variables with non-zero coefficients. The intersection of the variables selected by the two methods resulted in 7 common variables. Considering clinical expertise, total cholesterol (TC), which had relatively weak clinical significance, was excluded. Finally, 6 core predictors were confirmed: BMI, history of hyperlipidemia, Hcy, GA, sdLDL-C, and UHR. A collinearity test was performed on these six variables, and all variance inflation factors (VIFs) were less than 5, indicating no significant multicollinearity. The results of the multivariable logistic regression are shown in Supplementary Table S6, the collinearity test results in Supplementary Table S7, and the variable selection process of LASSO regression in Figures 7A,B.

### 3.9 Machine learning model evaluation and selection

Based on the six chosen core predictors (BMI, history of hyperlipidemia, Hcy, GA, sdLDL-C, and UHR), seven ML models were constructed on the training set (*n* = 1,917). Hyperparameters were optimized using grid search combined with five-fold cross-validation (optimal parameters are shown in Supplementary Table S8). The performance evaluation results of each model are summarized in Table 4.

**Table 4:** Performance evaluation of machine learning models on the training set and internal validation set.

Model	AUROC (95% CI)	Accuracy	Precision	Sensitivity	Specificity	F1 Score	Kappa	Youden’s J	PPV	NPV
Training set										
Logistic	0.672 (0.645–0.695)	0.673	0.551	0.191	0.920	0.284	0.133	0.111	0.551	0.690
Decision Tree	0.912 (0.899–0.923)	0.828	0.735	0.769	0.858	0.752	0.620	0.627	0.735	0.879
Random Forest	1.000 (1.000–1.000)	1.000	1.000	1.000	1.000	1.000	1.000	1.000	1.000	1.000
XGBoost	0.922 (0.911–0.933)	0.835	0.833	0.639	0.935	0.724	0.609	0.574	0.833	0.835
LightGBM	1.000 (0.999–1.000)	0.990	0.988	0.983	0.994	0.985	0.978	0.977	0.988	0.991
SVM	0.666 (0.639–0.689)	0.665	0.594	0.029	0.990	0.056	0.025	0.019	0.594	0.666
ANN	0.726 (0.702–0.748)	0.707	0.600	0.407	0.861	0.485	0.291	0.268	0.600	0.739
Validation set										
Logistic	0.654 (0.618–0.695)	0.678	0.567	0.199	0.923	0.294	0.144	0.121	0.567	0.693
Decision Tree	0.752 (0.716–0.787)	0.718	0.582	0.588	0.785	0.585	0.372	0.373	0.582	0.789
Random Forest	0.790 (0.758–0.823)	0.741	0.667	0.469	0.880	0.551	0.377	0.350	0.667	0.765
XGBoost	0.866 (0.839–0.890)	0.782	0.750	0.531	0.910	0.622	0.474	0.440	0.750	0.792
LightGBM	0.873 (0.850–0.895)	0.796	0.752	0.592	0.901	0.663	0.520	0.493	0.752	0.812
SVM	0.655 (0.618–0.695)	0.666	0.571	0.043	0.983	0.081	0.035	0.027	0.571	0.668
ANN	0.659 (0.622–0.700)	0.673	0.525	0.347	0.840	0.417	0.203	0.186	0.525	0.716

AUROC, Area Under the Receiver Operating Characteristic Curve; CI, Confidence Interval; PPV, Positive Predictive Value; NPV, Negative Predictive Value; Logistic, Logistic Regression; Decision Tree, Decision Tree; Random Forest, Random Forest; XGBoost, Extreme Gradient Boosting; LightGBM, Light Gradient Boosting Machine; SVM, Support Vector Machine; ANN, Artificial Neural Network.

**Figure 8:**
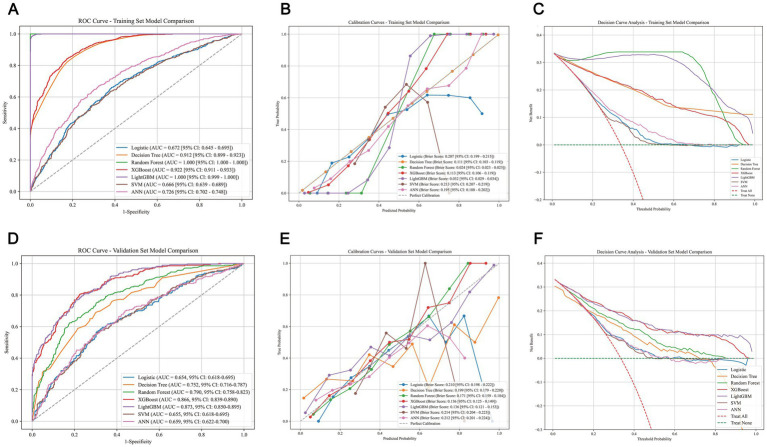
Assessment of model performance on the training set and validation set. **(A)** Receiver operating characteristic (ROC) curves on the training set. **(B)** Calibration curves on the training set. **(C)** Decision curve analysis (DCA) on the training set. **(D)** Receiver operating characteristic (ROC) curves on the validation cohort. **(E)** Calibration curves on the validation cohort. **(F)** Decision curve analysis (DCA) on the validation cohort. AUC, area under the curve; CI, confidence interval; Logistic, logistic regression; XGBoost, eXtreme Gradient Boosting; LightGBM, Light Gradient Boosting Machine; SVM, support vector machine; ANN, artificial neural network.

In the training set, both the RF and Light Gradient Boosting Machine (LightGBM) models exhibited near-perfect discrimination ability (AUC = 1.000), suggesting a potential risk of overfitting. The XGBoost model also achieved good performance (AUC = 0.922, 95% CI: 0.911–0.933, Figure 8A), with an accuracy of 0.835, sensitivity of 0.639, and specificity of 0.935. In the internal validation set, the generalization abilities of the models diverged. The XGBoost model showed the most robust performance, with an AUC of 0.866 (95% CI: 0.839–0.890, Figure 8D), accuracy of 0.782, sensitivity of 0.531, and specificity of 0.910. Although LightGBM achieved a slightly higher AUC (0.873), the difference in performance between the training and validation sets was larger, and its calibration ability was inferior to that of XGBoost. Calibration curve analysis (Figures 8B,E) showed that the predicted probabilities of XGBoost were in good agreement with the observed probabilities (Brier Score = 0.136). Calibration curve analysis (Figures 7B,E) showed good agreement between the predicted probabilities from XGBoost and the observed probabilities. Given that the incidence of the composite outcome in this study population was 33.8%, the Brier Score of the null model was 0.224 (i.e., incidence × [1 − incidence]), while the XGBoost model achieved a Brier Score of 0.136, representing a marked improvement over the null model and indicating that its calibration performance meets the basic requirements for clinical risk stratification. Decision curve analysis (Figures 8C,F) further demonstrated that the XGBoost model provided a stable and substantial net benefit across a range of clinically relevant decision thresholds.

After comprehensively comparing the discrimination, calibration, and clinical utility of every model, XGBoost was chosen as the final model for predicting the 180-day composite outcome risk in patients with HF, as it effectively controlled overfitting while demonstrating the best generalization ability.

### 3.10 Variable importance interpretation

**Figure 9:**
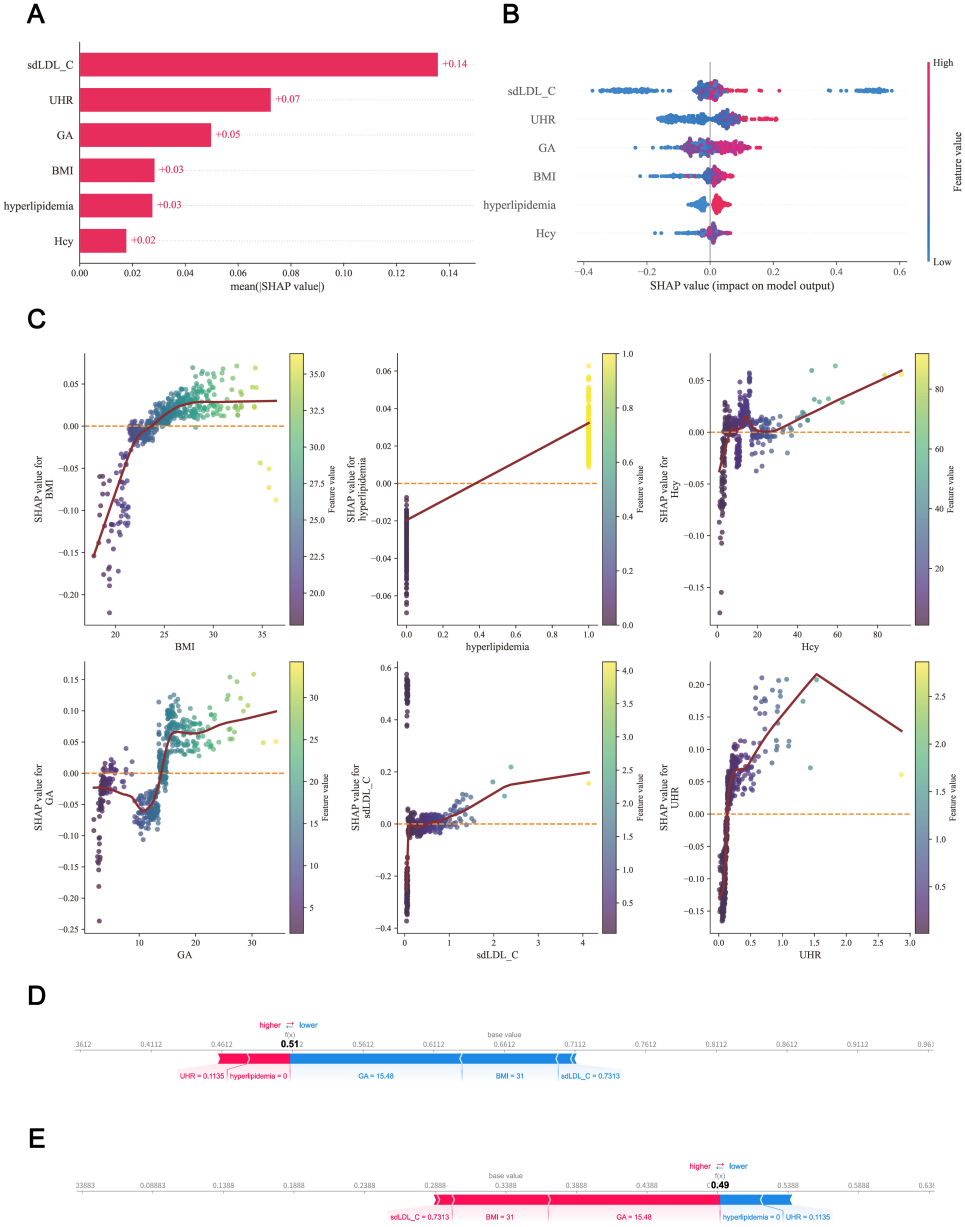
Interpretability analysis of the best-performing model (XGBoost, extreme gradient boosting). **(A)** Global feature importance bar plot based on SHapley additive explanations (SHAP) values. **(B)** Feature importance summary plot (beeswarm plot). **(C)** Feature dependence plots. **(D–G)** Individual prediction explanation force plots. SHAP, SHapley additive explanations; UHR, uric acid to high-density lipoprotein cholesterol ratio; GA, glycoalbumin; sdLDL-C, small dense low-density lipoprotein cholesterol; BMI, body mass index; Hcy, homocysteine.

To elucidate the decision-making logic of the optimal XGBoost model, the SHAP framework was used for model interpretability analysis. The global feature importance ranking (Figure 9A) showed that sdLDL-C was the feature contributing the most to the model output, followed by UHR, GA, BMI, history of hyperlipidemia, and Hcy. The SHAP beeswarm plot (Figure 9B) intuitively presented the direction of influence of each feature on the predicted risk: higher values of GA and BMI tended to increase the predicted risk of the composite outcome; a history of hyperlipidemia (coded as 1) positively drove the model output; while sdLDL-C, UHR, and Hcy exhibited nonlinear relations with the composite outcome risk. The SHAP dependence plots (Figure 9C) further revealed the complex relationships between key variables and the composite outcome risk. Decomposition of SHAP values for representative individual predictions in patients with HF (Figures 9D,E) further confirmed that for a low-risk prediction (Figure 9D, probability 0.51), GA (15.48 g/dL), BMI (31), and sdLDL-C (0.7313 mmol/L) provided significant protective effects, while UHR (0.1135) and the absence of hyperlipidemia contributed negatively. For an individual predicted to be at high risk (Figure 9E, probability 0.49), GA (15.48 g/dL) was the most significant negative driving factor.

In summary, the SHAP analysis clearly quantified the contribution of each feature to the XGBoost model’s predictions and revealed its decision logic. The model integrated a broad spectrum of indicators spanning metabolism, inflammation, and lipid profiles, and its decision patterns aligned with the multiple pathophysiological mechanisms underlying heart failure prognosis, substantially enhancing the model’s transparency and clinical interpretability.

### 3.11 Deployment of the prediction model as a web-based tool

**Figure 10:**
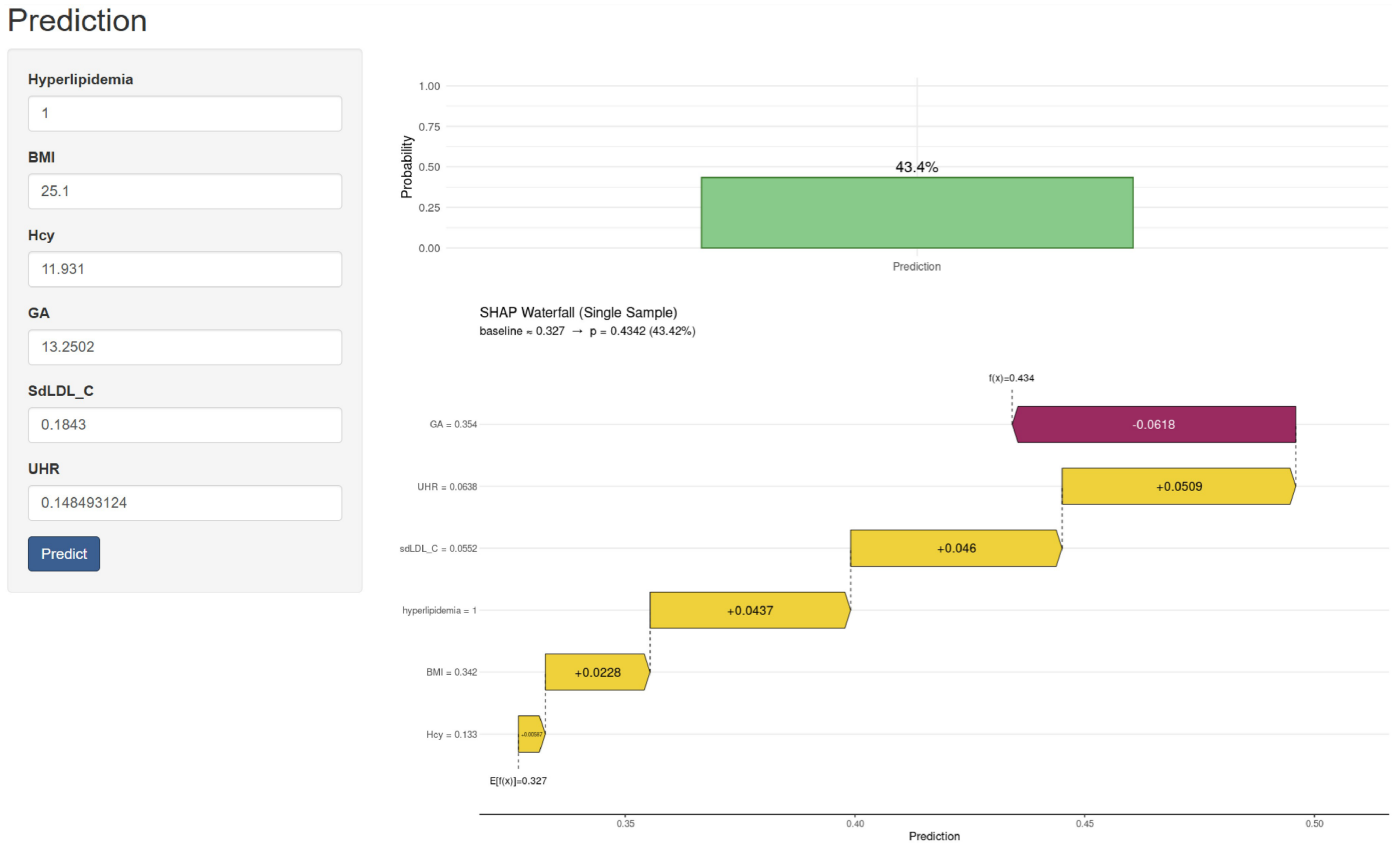
A web-based prediction tool for 180-day prognosis in heart failure. UHR, uric acid to high-density lipoprotein cholesterol ratio; GA, glycoalbumin; sdLDL-C, small dense low-density lipoprotein cholesterol; BMI, body mass index; Hcy, homocysteine.

Based on six selected variables, an online calculator was developed to predict the 180-day composite outcome risk in heart failure patients. Figure 10 displays the web deployment interface of the prediction model, which is accessible at the following URL: https://machine11.shinyapps.io/xgboostforUHR/.

It is important to emphasize that the findings of this study support an exploratory prognostic association between UHR and the composite outcome, as well as the preliminary feasibility of using machine learning for risk prediction in heart failure. However, the model has not yet undergone external validation. Therefore, the deployed online calculator is intended strictly for research purposes and should not be used for clinical decision-making at this stage.

## 4 Discussion

This study integrated UHR into an ML framework to systematically evaluate its predictive value for the short-term composite outcome in patients with HF and constructed an interpretable risk prediction model based on this indicator. We found that UHR was significantly and independently positively associated with the risk of the 180-day composite outcome in patients with HF, with a nonlinear dose–response relationship. In both the HFrEF and HFpEF subtypes, UHR maintained stable predictive efficacy and outperformed other composite lipid indices such as AIP, NHHR, and TyG. The XGBoost model, constructed based on six core variables (UHR, BMI, history of hyperlipidemia, Hcy, GA, and sdLDL-C), achieved an AUC of 0.866 in the internal validation set, with good calibration, and DCA demonstrated its high clinical net benefit for predicting the composite outcome. Interpretation of the model using the SHAP framework confirmed UHR as the second most important predictive feature after sdLDL-C and revealed nonlinear relationships between the variables and the composite outcome risk.

Baseline BNP and eGFR were broadly similar between the composite outcome group and the event-free group. Furthermore, the median urea-a marker reflecting volume status and renal perfusion-was identical between the two groups, and the between-group differences in median serum albumin and prealbumin were minimal, with hs-CRP also showing no significant difference. These indicators from multiple dimensions, including renal function, volume status, nutritional metabolism, and systemic inflammation, all point to a roughly balanced overall heart failure severity between the two groups at enrollment. This reduces the potential confounding effect of systematic bias in baseline disease severity on the association between UHR and the composite outcome. It also indirectly suggests that the risk information conveyed by UHR may be relatively independent of these classical heart failure severity markers, pointing toward a metabolic-inflammatory risk dimension.

Compared with existing studies, UHR, as a composite indicator integrating UA and HDL-C, has been preliminarily confirmed in previous research for its relation with the prognosis of cardiovascular diseases (13). Building on this foundation, our study is the first to introduce UHR as a core predictor into an ML model and evaluate its predictive contribution under multivariable interactions. The results confirmed that UHR has superior predictive efficacy for the composite outcome in HF compared with other composite indices (such as AIP and NHHR), and the strength of the relation was higher in the HFrEF subgroup than in the HFpEF subgroup. This difference may be related to the more pronounced systemic metabolic disturbances and inflammatory state in patients with HFrEF, and it also reflects the heterogeneity of the pathophysiological mechanisms underlying different HF subtypes (23–25).

Regarding the association between HF etiology and high-risk patients, our cohort data demonstrated that the proportion of ischaemic cardiomyopathy (ICM) was significantly higher in the composite outcome group than in the group without the composite outcome (51.46% vs. 38.73%), while patients with valvular heart disease (VHD) or hypertensive heart disease (HHD) had relatively lower event rates. This finding suggests that ischaemic etiology represents the high-risk subgroup with the poorest prognosis within this HF cohort, consistent with previous studies reporting adverse outcomes in ischaemic HF (26).

From a pathophysiological perspective, UHR reflects an imbalance between oxidative stress and inflammation, which is a central link in the development and progression of HF (27–29). UA is produced in increased amounts under conditions of ischemia and hypoxia and exacerbates myocardial injury and ventricular remodeling by activating the NLRP3 inflammasome and promoting the production of reactive oxygen species (14). HDL-C exerts cardiovascular protective effects through reverse cholesterol transport, antioxidant, anti-inflammatory, and endothelial protective functions (30). In the state of HF, HDL-C levels are often decreased and its function impaired (31, 32), while UA levels are elevated due to factors such as reduced renal perfusion and tissue hypoxia (33, 34). By combining these two, UHR can more comprehensively capture the degree of disturbance in the metabolic-inflammatory network of patients with HF. The sensitivity analysis using joint stratification of UA and HDL-C in this study further confirmed that the prognostic value of UHR is not driven by either single component alone. Neither isolated high UA nor isolated low HDL-C alone produced a risk increment comparable to that when both conditions coexist, which, from a clinical perspective, directly supports the mechanistic plausibility of UHR as a composite indicator of oxidative stress–antioxidant imbalance. In this study, a sex difference was observed in the association between UHR and the composite outcome among HFpEF patients, with the association being substantially stronger in women than in men. From a biological perspective, this phenomenon may be explained by the following mechanisms: the loss of estrogen protection in postmenopausal women leads to a marked elevation in uric acid levels, accompanied by alterations in the composition and antioxidant function of HDL-C particles, thereby making the oxidative stress–antioxidant imbalance represented by UHR more prominent in women (35, 36). In addition, female HFpEF patients more frequently present with central obesity and microvascular dysfunction, which are closely linked to uric acid-mediated endothelial injury and HDL dysfunction (37, 38). However, given the limited sample size of the subgroups in this study, this sex-specific hypothesis still requires independent validation in prospective studies.

In terms of model construction, the XGBoost algorithm used in this study, through gradient boosting and regularization strategies, effectively controlled overfitting while maintaining high predictive performance (39), and its comprehensive performance was superior to other models such as random forest and LightGBM. Compared with traditional logistic regression, XGBoost can automatically capture nonlinear relationships and interactions among variables, better aligning with the complex pathophysiological processes of HF (40, 41). Although a purely random split rather than stratified sampling was used to partition the training and validation sets, the baseline characteristics of the two sets were comparable, and the composite outcome incidence was nearly identical (33.9% vs. 33.8%; Supplementary Tables S2, S3), indicating that the random partition did not introduce meaningful imbalance. The indicators used in the model are all routine clinical tests, ensuring its potential for promotion and application in medical institutions at all levels. BMI, as a bidirectional indicator of nutritional status and metabolic load, when excessively high, exacerbates ventricular remodeling by increasing volume load, promoting the release of pro-inflammatory factors, and inducing insulin resistance (42). A history of hyperlipidemia represents the cumulative atherosclerotic burden of long-term abnormal lipid metabolism, and the coronary artery disease, lipotoxic myocardial injury, and residual inflammatory risk mediated by it are important bases for the development and progression of HF (43, 44). Hcy accelerates atherosclerosis and myocardial fibrosis by directly damaging the vascular endothelium and activating oxidative stress and inflammatory pathways (45, 46). GA, as a sensitive indicator of short-term blood glucose fluctuations, its elevation reflects stress hyperglycemia, which directly damages endothelial function and increases myocardial stiffness through the formation of advanced glycation end products (47). sdLDL-C, as the most atherogenic LDL subtype, plays a central role in coronary atherosclerosis and microcirculatory dysfunction due to its stronger endothelial penetrability and pro-inflammatory properties (48, 49). These five indicators systematically capture the complex nature of HF as a multisystem metabolic-inflammatory disorder from irreplaceable pathological links, including metabolic load, vascular damage, glycemic disturbance, and lipid quality.

The clinical significance of this study lies in the fact that it not only validates the value of UHR as a prognostic marker for heart failure but also integrated it into a modern ML framework, ultimately transforming it into a convenient online tool. Clinicians or researchers can access this tool, input UHR and other routine indicators, and obtain an individualized risk assessment result for the 180-day composite outcome, thereby enabling the optimization of risk stratification and intervention strategies. This translational pathway from biomarker to clinical tool provides a new approach for the application of precision medicine in HF management.

This study has several limitations. First, the single-center retrospective design may introduce selection bias and cannot establish causality. Second, UHR was based on a single baseline measurement and did not capture longitudinal changes during follow-up. Third, the model was not externally validated in an independent cohort. Fourth, detailed medication data and treatment regimens were not included. An E-value sensitivity analysis indicated that an unmeasured confounder would need to be associated with both UHR and the composite outcome at a risk ratio of approximately 1.48 to fully explain away the observed association, which provides some reassurance regarding the robustness of the core findings; however, this limitation cannot be entirely eliminated. Fifth, the outcome was limited to all-cause death and HF rehospitalisation and did not include events such as myocardial infarction or stroke. Sixth, the assessment of echocardiographic parameters in this study relied primarily on LVEF and did not incorporate other multidimensional echocardiographic measures. Future prospective studies need to include comprehensive medication records, detailed echocardiographic data, and serial biomarker measurements to address these limitations. Systematically collecting multidimensional echocardiographic indicators related to cardiac remodeling and integrating them into prediction models represents an important direction for enhancing the pathophysiological coverage and clinical interpretability of the models.

In conclusion, this study confirms that UHR is an independent predictor of the short-term composite outcome risk in patients with HF. The XGBoost model based on UHR and routine clinical indicators demonstrates good predictive performance and clinical utility, and has the potential to become a new tool for optimizing risk stratification and individualized management of HF. The online deployment of the model further lowers the barrier to its application, providing convenient support for research risk assessment.

## 5 Conclusion

This study confirms that UHR is an independent risk factor for the 180-day composite outcome in patients with heart failure, and its predictive performance is superior to other composite lipid indices such as AIP and NHHR. By integrating UHR and five routine clinical indicators (BMI, history of hyperlipidemia, Hcy, GA, sdLDL-C), the predictive model constructed using the XGBoost algorithm demonstrated good discriminative ability (AUC = 0.866) and calibration in internal validation. SHAP analysis enhanced the clinical interpretability of the model. The model has been deployed as an online research demonstration platform. Pending external validation, it may serve as an exploratory tool for short-term risk assessment. This tool facilitates rapid individualized risk stratification for research purposes and holds promise as an exploratory instrument for optimizing short-term prognosis assessment in patients with heart failure, pending external validation.

## Data Availability

The data analyzed in this study is subject to the following licenses/restrictions: Due to the fact that the dataset is sourced from retrospective data from Beijing Anzhen Hospital, Capital Medical University, the dataset analyzed during this study is not publicly available, but can be obtained from the corresponding author upon reasonable request. Requests to access these datasets should be directed to nanjingzhu0885@126.com.

## Glossary

AIP: Atherogenic index of plasma
Alb: Albumin
ALP: Alkaline phosphatase
ALT: Alanine aminotransferase
AST: Aspartate aminotransferase
BMI: Body mass index
BNP: Brain natriuretic peptide
Ca: Calcium
ChE: Cholinesterase
CI: Confidence interval
CK: Creatine kinase
Cr: Creatinine
CysC: Cystatin C
DB: Direct bilirubin
eGFR: estimated glomerular filtration rate
GA: Glycoalbumin
GGT: Gamma-glutamyl transferase
Glu: Glucose
Hcy: Homocysteine
HDL-C: High-density lipoprotein cholesterol
HFrEF: Heart failure with reduced ejection fraction
HFpEF: Heart failure with preserved ejection fraction
hs-CRP: High-sensitivity C-reactive protein
IQR: Interquartile range
LD: Lactate dehydrogenase
LDL-C: Low-density lipoprotein cholesterol
LPa: Lipoprotein(a)
NHHR: Non-high-density lipoprotein cholesterol to high-density lipoprotein cholesterol ratio
OR: Odds ratio
PA: Prealbumin
sdLDL-C: Small dense low-density lipoprotein cholesterol
SE: Standard error
SHAP: SHapley Additive exPlanations
TB: Total bilirubin
TBA: Total bile acid
TC: Total cholesterol
TG: Triglycerides
TyG: Triglyceride-glucose index
UA: Uric acid
UHR: Uric acid to high-density lipoprotein cholesterol ratio

## References

[1] Wańczura P Aebisher D Wiśniowski M Kos M Bukowski H Golicki D . Cost-utility analysis of 3-month telemedical intervention for heart failure patients: a preliminary study from Poland. Health Care (Don Mills). (2024) 12:1360. doi: 10.3390/healthcare12131360, 38998893 PMC11240905

[2] Naito T Nakamura K Abe Y Watanabe H Sakuragi S Katayama Y . Prevalence of transthyretin amyloidosis among heart failure patients with preserved ejection fraction in Japan. ESC Heart Fail. (2023) 10:1896–906. doi: 10.1002/ehf2.14364, 39868751 PMC10192249

[3] Dunlay SM Roger VL Redfield MM. Epidemiology of heart failure with preserved ejection fraction. Nat Rev Cardiol. (2017) 14:591–602. doi: 10.1038/nrcardio.2017.65, 28492288

[4] Borlaug BA Sharma K Shah SJ Ho JE. Heart failure with preserved ejection fraction: JACC scientific statement. J Am Coll Cardiol. (2023) 81:1810–34. doi: 10.1016/j.jacc.2023.01.049, 37137592

[5] Abdin A Anker SD Butler J Coats AJS Kindermann I Lainscak M . ‘Time is prognosis’ in heart failure: time-to-treatment initiation as a modifiable risk factor. ESC Heart Fail. (2021) 8:4444–53. doi: 10.1002/ehf2.13646, 34655282 PMC8712849

[6] Zannad F Reddy YNV Barash I Anstrom KJ Bonaca MP Borentain M . Effect of vericiguat on total heart failure events in compensated outpatients with HFrEF: insights from VICTOR. J Am Coll Cardiol. (2025) 86:2471–91. doi: 10.1016/j.jacc.2025.08.051, 40892609

[7] Gallo G Rubattu S Volpe M. Mitochondrial dysfunction in heart failure: from pathophysiological mechanisms to therapeutic opportunities. Int J Mol Sci. (2024) 25:2667. doi: 10.3390/ijms25052667, 38473911 PMC10932393

[8] Li G Zhao H Cheng Z Liu J Guo Y. Single-cell transcriptomic profiling of heart reveals ANGPTL4 linking fibroblasts and angiogenesis in heart failure with preserved ejection fraction. J Adv Res. (2025) 68:215–30. doi: 10.1016/j.jare.2024.02.006, 38346487 PMC11785561

[9] Sun Y Ji H Sun W An X Lian F. Triglyceride glucose (TyG) index: a promising biomarker for diagnosis and treatment of different diseases. Eur J Intern Med. (2025) 131:3–14. doi: 10.1016/j.ejim.2024.08.026, 39510865

[10] Lai Y Lin C Liu X Liu Y Cai H Zhao N . Association of triglyceride-glucose index trajectories with the risk of worsening heart failure in elderly patients with chronic heart failure and type 2 diabetes: a competing risk analysis. Cardiovasc Diabetol. (2025) 24:131. doi: 10.1186/s12933-025-02687-8, 40119388 PMC11929357

[11] Fu Y Song G Wu S Ma L Bao X Liu X . The combined association of the neutrophil percentage-to-albumin ratio (NPAR) and the uric acid-to-high-density lipoprotein cholesterol ratio (UHR) with adverse cardiac events in patients with chronic heart failure: a retrospective cohort study. Lipids Health Dis. (2025) 25:1. doi: 10.1186/s12944-025-02807-z, 41275181 PMC12764124

[12] Xie Y Huang K Zhang X Wu Z Wu Y Chu J . Association of serum uric acid-to-high-density lipoprotein cholesterol ratio with non-alcoholic fatty liver disease in American adults: a population-based analysis. Front Med. (2023) 10:1164096. doi: 10.3389/fmed.2023.1164096, 37256087 PMC10225665

[13] Li Z Liu Q Yao Z. The serum uric acid-to-high-density lipoprotein cholesterol ratio is a predictor for all-cause and cardiovascular disease mortality: a cross-sectional study. Front Endocrinol. (2024) 15:1417485. doi: 10.3389/fendo.2024.1417485, 39345882 PMC11427315

[14] Andretto de Mattos B Tomas-Grau RH Alves Fernandes TA González-Lizárraga F Tourville A Ciss I . Uric acid, the end-product of purine metabolism, mitigates tau-related abnormalities: comparison with DOT, a non-antibiotic oxytetracycline derivative. Biomolecules. (2025) 15:941. doi: 10.3390/biom15070941, 40723813 PMC12292136

[15] Sirtori CR Corsini A Ruscica M. The role of high-density lipoprotein cholesterol in 2022. Curr Atheroscler Rep. (2022) 24:365–77. doi: 10.1007/s11883-022-01012-y, 35274229 PMC8913032

[16] de Oliveira WPC Freitas FR Costa MT Silva AO de Cleva R Kalil Filho R . Cholesterol transfer to high-density lipoprotein in obesity and the effects of weight loss after bariatric surgery. Clin Obes. (2024) 14:e12688. doi: 10.1111/cob.12688, 38943556

[17] Liu P Li J Yang L Zhang Z Zhao H Zhao N . Association between cumulative uric acid to high-density lipoprotein cholesterol ratio and the incidence and progression of chronic kidney disease. Front Endocrinol. (2023) 14:1269580. doi: 10.3389/fendo.2023.1269580, 38155948 PMC10753577

[18] Kahaer M Zhu Y He Y Chen W Yang D du M . A comprehensive study on the uric acid to high-density cholesterol ratio influencing cardiometabolic-based chronic disease in China. Front Nutr. (2026) 13:1723098. doi: 10.3389/fnut.2026.1723098, 41798840 PMC12962957

[19] Wang J Duan S Li H Wu S Zhao P Zhu T . Development and validation of a prognostic nomogram for post-transcatheter aortic valve replacement heart failure hospitalization in patients with concurrent symptomatic aortic stenosis and heart failure with preserved ejection fraction: a multicenter study. J Am Heart Assoc. (2026) 15:e046606. doi: 10.1161/JAHA.125.046606, 41804883 PMC13055727

[20] Wang Y Wang Y Luan Y Hao M Yin W Wu B . Multimodal data-driven explainable prognostic model for major adverse cardiovascular events prediction in patients with unstable angina and heart failure with preserved ejection fraction: multicenter, cross-regional cohort study. J Med Internet Res. (2025) 27:e78402. doi: 10.2196/78402, 41397298 PMC12705130

[21] Wang J Zhu J Li H Wu S Li S Yao Z . Multimodal visualization and explainable machine learning-driven markers enable early identification and prognosis prediction for symptomatic aortic stenosis and heart failure with preserved ejection fraction after transcatheter aortic valve replacement: multicenter cohort study. J Med Internet Res. (2025) 27:e70587. doi: 10.2196/70587, 40310672 PMC12082054

[22] Ruan H Duan S He L Wang Y Yao Z Pan L . The incremental prognostic value of incorporating the triglyceride-glucose index into the traditional cardiovascular risk factors for the long-term prognosis in ischemic cardiomyopathy patients with HFpEF following coronary artery bypass grafting: a multicenter cohort study. J Atheroscler Thromb. (2025) 32:1251–67. doi: 10.5551/jat.65654, 40222904 PMC12504032

[23] Suciu IM Mateoc-Sîrb T Luca CT Timar B Gaiță D. Patterns in prescribing and predictors of SGLT2 inhibitor administration in patients with heart failure and acute myocardial infarction: a real-world retrospective cohort study. J Clin Med. (2026) 15:1056. doi: 10.3390/jcm15031056, 41682737 PMC12898543

[24] Méndez-Fernández A Fernández-Mora Á Bernal-Ramírez J Alves-Figueiredo H Nieblas B Salazar-Ramírez F . Distinguishing pathophysiological features of heart failure with reduced and preserved ejection fraction: a comparative analysis of two mouse models. J Physiol. (2024). doi: 10.1113/JP286410, 39018163

[25] Shuaishuai D Jingyi L Zhiqiang Z Guanwei F. Sex differences and related estrogenic effects in heart failure with preserved ejection fraction. Heart Fail Rev. (2023) 28:937–48. doi: 10.1007/s10741-022-10274-2, 36190606

[26] Lee J-G Beom JW Choi JH Kim SY Kim KS Joo SJ. Pseudonormal or restrictive filling pattern of left ventricle predicts poor prognosis in patients with ischemic heart disease presenting as acute heart failure. J Cardiovasc Imaging. (2018) 26:217–25. doi: 10.4250/jcvi.2018.26.e22, 30607389 PMC6310756

[27] Vijay K Neuen BL Lerma EV. Heart failure in patients with diabetes and chronic kidney disease: challenges and opportunities. Cardiorenal Med. (2022) 12:1–10. doi: 10.1159/000520909, 34802000

[28] Tang Y Liu J Zhang J Zhu Y Zhou J. Association of serum uric acid-to-high-density lipoprotein cholesterol ratio with obstructive sleep apnea: a cross-sectional study. Lipids Health Dis. (2025) 24:188. doi: 10.1186/s12944-025-02604-8, 40413493 PMC12102946

[29] Wang J Wang Y Niu Y Wang J Shi L Guo Y . Association between uric acid to high-density lipoprotein cholesterol ratio (UHR) and female infertility: insights from a cross-sectional study. Eur J Med Res. (2025) 30:854. doi: 10.1186/s40001-025-03084-3, 40999482 PMC12465579

[30] Kosmas CE Sourlas A Guzman E Kostara CE. Environmental factors modifying HDL functionality. Curr Med Chem. (2022) 29:1687–701. doi: 10.2174/0929867328666210714155422, 34269662

[31] Hui N Barter PJ Ong K-L Rye K-A. Altered HDL metabolism in metabolic disorders: insights into the therapeutic potential of HDL. Clin Sci. (2019) 133:2221–35. doi: 10.1042/CS20190873 31722013

[32] Kuburovic V Vekic J Zeljkovic A Carrie A Kotur-Stevuljevic J Bojanin D . The usefulness of advanced lipid and oxidative stress testing for diagnosis and management of low HDL-cholesterol phenotype: a case report. Clin Biochem. (2017) 50:1323–5. doi: 10.1016/j.clinbiochem.2017.06.007, 28648695

[33] Wu S Xue W Yu H Yu H Shi Z Wang L . Serum uric acid levels and health outcomes in CKD: a prospective cohort study. Nephrol Dial Transplant. (2024) 39:511–9. doi: 10.1093/ndt/gfad201 37698875

[34] Freilich M Arredondo A Zonnoor SL McFarlane IM. Elevated serum uric acid and cardiovascular disease: a review and potential therapeutic interventions. Cureus. (2022) 14:e23582. doi: 10.7759/cureus.23582 35494989 PMC9045796

[35] Zhou X. Relationship between serum uric acid levels and metabolism associated fatty liver disease in postmenopausal women based on NHANES 2017-2020. Sci Rep. (2025) 15:8944. doi: 10.1038/s41598-025-93738-3, 40089555 PMC11910611

[36] Wan H Zhang K Wang Y Chen Y Zhang W Xia F . The associations between gonadal hormones and serum uric acid levels in men and postmenopausal women with diabetes. Front Endocrinol. (2020) 11:55. doi: 10.3389/fendo.2020.00055, 32153501 PMC7044188

[37] Hafiane A Favari E Bortnick AE. Measures of high-density lipoprotein function in men and women with severe aortic stenosis. Lipids Health Dis. (2022) 21:48. doi: 10.1186/s12944-022-01653-7, 35643498 PMC9148512

[38] Wu G Liu J Ma G Wei Q Song X. Hyperuricemia facilitates uric acid-mediated vascular endothelial cell damage by inhibiting mitophagy. Cell Biochem Biophys. (2025) 83:811–21. doi: 10.1007/s12013-024-01512-5, 39340591 PMC11870927

[39] Lai Y Lin P Lin F Chen M Lin C Lin X . Identification of immune microenvironment subtypes and signature genes for alzheimer’s disease diagnosis and risk prediction based on explainable machine learning. Front Immunol. (2022) 13:1046410. doi: 10.3389/fimmu.2022.1046410, 36569892 PMC9773397

[40] Lin L Xie Y Lin Z Lin C Yang Y. Machine learning for predicting metabolic-associated fatty liver disease including NHHR: a cross-sectional NHANES study. PLoS One. (2025) 20:e0319851. doi: 10.1371/journal.pone.0319851, 40100868 PMC11918377

[41] Tu JV. Advantages and disadvantages of using artificial neural networks versus logistic regression for predicting medical outcomes. J Clin Epidemiol. (1996) 49:1225–31. doi: 10.1016/S0895-4356(96)00002-9, 8892489

[42] Gutin I. In BMI we trust: reframing the body mass index as a measure of health. Soc Theory Health. (2018) 16:256–71. doi: 10.1057/s41285-017-0055-0, 31007613 PMC6469873

[43] Altschmiedová T Vaclová M Vráblík M. Diagnosis of familial hypercholesterolaemia on first sight? The role of the ophthalmologist in identifying patients with familial hypercholesterolaemia. Ces Slov Oftalmol. (2019) 74:127–31. doi: 10.31348/2018/1/1-4-2018 30913887

[44] Grundy SM. 2018 AHA/ACC/AACVPR/AAPA/ABC/ACPM/ADA/AGS/APhA/ASPC/NLA/PCNA guideline on the management of blood cholesterol: a report of the American College of Cardiology/American Heart Association task force on clinical practice guidelines. Circulation. (2019) 139:e1082–143. doi: 10.1161/cir.0000000000000625, 30586774 PMC7403606

[45] Balint B Jepchumba VK Guéant J-L Guéant-Rodriguez R-M. Mechanisms of homocysteine-induced damage to the endothelial, medial and adventitial layers of the arterial wall. Biochimie. (2020) 173:100–6. doi: 10.1016/j.biochi.2020.02.012 32105811

[46] Zhang S Lv Y Luo X Weng X Qi J Bai X . Homocysteine promotes atherosclerosis through macrophage pyroptosis via endoplasmic reticulum stress and calcium disorder. Mol Med. (2023) 29:73. doi: 10.1186/s10020-023-00656-z, 37308812 PMC10262416

[47] Cohen MP Clements RS Cohen JA Shearman CW. Glycated albumin promotes a generalized vasculopathy in the db/db mouse. Biochem Biophys Res Commun. (1996) 218:72–5. doi: 10.1006/bbrc.1996.0014, 8573179

[48] Ma X Wang Q Hu X Wang X Zhao Y Liu X . Association of sdLDL-C with incident carotid plaques with stable and vulnerable morphology: a prospective cohort study. Stroke. (2024) 55:576–85. doi: 10.1161/STROKEAHA.123.045601, 38214156

[49] Santos HO Earnest CP Tinsley GM Izidoro LFM Macedo RCO. Small dense low-density lipoprotein-cholesterol (sdLDL-C): analysis, effects on cardiovascular endpoints and dietary strategies. Prog Cardiovasc Dis. (2020) 63:503–9. doi: 10.1016/j.pcad.2020.04.009, 32353373

