# Supplementary material for: Predicting short-term composite outcome risk in heart failure patients using a machine learning model incorporating UHR: a retrospective cohort study
Source: Front Nutr. 2026 Jun 1;13:1846081. doi: 10.3389/fnut.2026.1846081 (PMC13265350; doi:10.3389/fnut.2026.1846081)
Supplement: Supplementary file 1 [file Table_1.docx]

**Supplementary materials (merged version)**

**Supplementary Table**

| **Supplementary Table S1. Missing Ratio of Variables in the Study** | |
| --- | --- |
| Variable | Missing Ratio |
| Outcome | 0 |
| LVEF | 0 |
| Ischaemic cardiomyopathy | 0 |
| Valvular heart disease | 0 |
| Dilated cardiomyopathy | 0 |
| Hypertensive heart disease | 0 |
| Other | 0 |
| sex | 0 |
| hypertension | 0 |
| diabetes | 0 |
| hyperlipidemia | 0 |
| age | 0 |
| BMI | 0 |
| Cr | 0 |
| PA | 0 |
| Hcy | 0 |
| hsCRP | 0 |
| ALT | 0 |
| AST | 0 |
| Alb | 0.002557545 |
| TB | 0.006576544 |
| DB | 0.004018999 |
| ALP | 0 |
| GGT | 0 |
| TBA | 0.002557545 |
| ChE | 0.002557545 |
| Urea | 0 |
| UA | 0 |
| Glu | 0 |
| CK | 0.010230179 |
| LD | 0.002922908 |
| TG | 0 |
| TC | 0 |
| HDL | 0 |
| LDL | 0 |
| Ca | 0 |
| GA | 0 |
| eGFR | 0.001826818 |
| LPa | 0.001826818 |
| sdLDL-C | 0 |
| CysC | 0 |
| BNP | 0 |
| AIP | 0 |
| UHR | 0 |
| NHHR | 0 |
| TyG | 0 |

Abbreviations: IQR, interquartile range; HFrEF, Heart Failure with reduced Ejection Fraction; HFpEF, Heart Failure with preserved Ejection Fraction; BMI, body mass index; Cr, creatinine; PA, prealbumin; Hcy, homocysteine; hs-CRP, high-sensitivity C-reactive protein; ALT, alanine aminotransferase; AST, aspartate aminotransferase; Alb, albumin; TB, total bilirubin; DB, direct bilirubin; ALP, alkaline phosphatase; GGT, gamma-glutamyl transferase; TBA, total bile acid; ChE, cholinesterase; UA, uric acid; Glu, glucose; CK, creatine kinase; LD, lactate dehydrogenase; TG, triglycerides; TC, total cholesterol; HDL-C, high-density lipoprotein cholesterol; LDL-C, low-density lipoprotein cholesterol; Ca, calcium; GA, glycoalbumin; eGFR, estimated glomerular filtration rate; LPa, lipoprotein(a); sdLDL-C, small dense low-density lipoprotein cholesterol; CysC, cystatin C; BNP, brain natriuretic peptide; AIP, atherogenic index of plasma; UHR, uric acid to high-density lipoprotein cholesterol ratio; NHHR, non-high-density lipoprotein cholesterol to high-density lipoprotein cholesterol ratio; TyG, triglyceride-glucose index; LVEF, Left Ventricular Ejection Fraction.

| **Supplementary Table S2. Baseline Characteristics of the Training Set** | | | | |
| --- | --- | --- | --- | --- |
|  |  |  |  |  |
| Variables | Total(n=1917) | No composite outcome(n=1268) | Composite outcome(n=649) | *p* |
| HFpEF(%) | 1286 (67.08) | 832 (65.62) | 454 (69.95) |  |
| HFrEF(%) | 631 (32.92) | 436 (34.38) | 195 (30.05) |  |
| LVEF (median [IQR]) | 59.00 [36.00, 64.00] | 59.00 [36.00, 64.00] | 60.00 [37.00, 65.00] | 0.127 |
| Causes of heart failure, n (%) | | | | |
| Ischaemic cardiomyopathy (%) | 842 (43.95) | 499 (39.38) | 343 (52.85) | <0.001 |
| Valvular heart disease (%) | 408 (21.29) | 312 (24.63) | 96 (14.79) | <0.001 |
| Dilated cardiomyopathy (%) | 347 (18.11) | 226 (17.84) | 121 (18.64) | 0.71 |
| Hypertensive heart disease (%) | 279 (14.56) | 193 (15.23) | 86 (13.25) | 0.273 |
| Other (%) | 45 ( 2.35) | 35 ( 2.76) | 10 ( 1.54) | 0.131 |
| age (median [IQR]) | 62.00 [53.00, 70.00] | 62.00 [53.00, 70.00] | 62.00 [54.00, 70.00] | 0.302 |
| BMI (median [IQR]) | 25.10 [22.80, 27.20] | 24.80 [22.50, 27.00] | 25.60 [23.70, 27.70] | <0.001 |
| sex (%) |  |  |  | 0.074 |
| Female | 535 (27.91) | 371 (29.26) | 164 (25.27) |  |
| Male | 1382 (72.09) | 897 (70.74) | 485 (74.73) |  |
| hypertension (%) | 935 (48.77) | 598 (47.16) | 337 (51.93) | 0.054 |
| diabetes (%) | 677 (35.32) | 420 (33.12) | 257 (39.60) | 0.006 |
| hyperlipidemia (%) | 1036 (54.04) | 629 (49.61) | 407 (62.71) | <0.001 |
| Cr (median [IQR])，(μmol/L) | 79.20 [64.80, 98.90] | 78.70 [63.27, 98.55] | 80.60 [67.70, 99.70] | 0.025 |
| PA (median [IQR]), (g/L) | 0.20 [0.10, 0.25] | 0.20 [0.09, 0.25] | 0.20 [0.12, 0.25] | 0.117 |
| Hcy (median [IQR])，(μmol/L) | 11.93 [7.08, 16.00] | 11.40 [6.39, 15.42] | 12.88 [8.55, 16.79] | <0.001 |
| hs-CRP (median [IQR])，(mg/L) | 2.96 [0.67, 20.00] | 3.01 [0.64, 20.00] | 2.90 [0.73, 20.00] | 0.99 |
| ALT (median [IQR])，(U/L) | 18.00 [12.00, 29.00] | 18.00 [12.00, 29.00] | 18.50 [13.00, 28.00] | 0.167 |
| AST (median [IQR])，(U/L) | 19.76 [15.36, 25.92] | 19.20 [14.40, 25.92] | 20.80 [16.64, 26.00] | 0.001 |
| Alb (median [IQR])，(g/L) | 41.50 [37.10, 44.90] | 41.30 [36.50, 44.73] | 41.90 [38.00, 45.20] | 0.005 |
| TB (median [IQR])，(μmol/L) | 12.60 [8.57, 18.30] | 12.80 [8.42, 18.40] | 12.28 [8.72, 18.12] | 0.908 |
| DB (median [IQR])，(μmol/L) | 3.92 [2.50, 5.99] | 3.92 [2.48, 6.04] | 3.94 [2.60, 5.87] | 0.533 |
| ALP (median [IQR])，(U/L) | 78.00 [56.00, 115.00] | 78.00 [55.00, 116.00] | 78.00 [59.00, 113.00] | 0.726 |
| GGT (median [IQR])，(U/L) | 37.44 [16.54, 240.96] | 34.56 [15.36, 240.96] | 41.60 [18.72, 261.04] | 0.018 |
| TBA (median [IQR])，(μmol/L) | 3.90 [1.80, 6.60] | 3.70 [1.80, 6.20] | 4.30 [1.80, 7.10] | 0.009 |
| ChE (median [IQR])，(kU/L) | 6.30 [3.50, 8.20] | 6.30 [3.40, 8.10] | 6.40 [3.90, 8.30] | 0.314 |
| Urea (median [IQR])，(mmol/L) | 6.76 [5.20, 9.03] | 6.78 [5.16, 8.91] | 6.74 [5.22, 9.19] | 0.488 |
| UA (median [IQR])，(μmol/L) | 324.33 [248.62, 411.06] | 308.65 [227.48, 390.66] | 365.40 [286.96, 443.10] | <0.001 |
| Glu (median [IQR])，(mmol/L) | 5.64 [4.77, 7.14] | 5.59 [4.70, 6.98] | 5.75 [4.92, 7.32] | 0.007 |
| CK (median [IQR])，(U/L) | 65.00 [40.00, 105.00] | 65.00 [40.00, 110.00] | 63.00 [40.00, 100.00] | 0.178 |
| LD (median [IQR])，(U/L) | 181.00 [148.00, 227.00] | 180.00 [147.00, 227.02] | 182.00 [149.00, 225.00] | 0.753 |
| TG (median [IQR])，(mmol/L) | 1.32 [0.82, 2.14] | 1.31 [0.83, 2.08] | 1.34 [0.81, 2.21] | 0.468 |
| TC (median [IQR])，(mmol/L) | 3.38 [2.23, 4.25] | 3.47 [2.23, 4.42] | 3.25 [2.24, 4.03] | 0.001 |
| HDL-C (median [IQR])，(mmol/L) | 0.99 [0.68, 1.22] | 1.03 [0.68, 1.28] | 0.91 [0.68, 1.12] | <0.001 |
| LDL-C (median [IQR])，(mmol/L) | 1.81 [0.86, 2.55] | 1.83 [0.84, 2.64] | 1.72 [0.90, 2.36] | 0.053 |
| Ca (median [IQR])，(mmol/L) | 2.29 [2.07, 2.41] | 2.29 [2.05, 2.41] | 2.30 [2.09, 2.41] | 0.558 |
| GA (median [IQR])，(g/dl) | 13.25 [8.79, 15.49] | 12.88 [7.27, 14.82] | 14.10 [10.88, 16.55] | <0.001 |
| eGFR (median [IQR])，(mL/min/1.73m^2) | 79.95 [56.09, 95.78] | 79.50 [54.16, 95.97] | 80.95 [57.14, 94.57] | 0.913 |
| LPa (median [IQR])，nmol/L | 22.20 [7.90, 63.90] | 21.10 [7.89, 60.73] | 24.20 [8.00, 70.60] | 0.236 |
| sdLDL-C (median [IQR])，mmol/L | 0.18 [0.06, 0.56] | 0.17 [0.06, 0.53] | 0.21 [0.06, 0.61] | 0.004 |
| CysC (median [IQR])，mg/L | 1.15 [0.51, 1.53] | 1.11 [0.42, 1.49] | 1.21 [0.86, 1.63] | <0.001 |
| BNP (median [IQR])，pg/ml | 130.00 [44.00, 481.00] | 125.65 [44.00, 439.50] | 146.00 [42.00, 564.00] | 0.147 |
| AIP (median [IQR]) | 1.21 [0.73, 1.71] | 1.16 [0.71, 1.66] | 1.30 [0.82, 1.77] | <0.001 |
| UHR (median [IQR]) | 0.15 [0.10, 0.23] | 0.13 [0.09, 0.21] | 0.17 [0.13, 0.27] | <0.001 |
| NHHR (median [IQR]) | 2.41 [1.74, 3.38] | 2.36 [1.71, 3.28] | 2.52 [1.78, 3.53] | 0.01 |
| TyG (median [IQR]) | 4.68 [4.40, 4.98] | 4.67 [4.41, 4.96] | 4.70 [4.40, 5.02] | 0.065 |

Abbreviations: IQR, interquartile range; HFrEF, Heart Failure with reduced Ejection Fraction; HFpEF, Heart Failure with preserved Ejection Fraction; BMI, body mass index; Cr, creatinine; PA, prealbumin; Hcy, homocysteine; hs-CRP, high-sensitivity C-reactive protein; ALT, alanine aminotransferase; AST, aspartate aminotransferase; Alb, albumin; TB, total bilirubin; DB, direct bilirubin; ALP, alkaline phosphatase; GGT, gamma-glutamyl transferase; TBA, total bile acid; ChE, cholinesterase; UA, uric acid; Glu, glucose; CK, creatine kinase; LD, lactate dehydrogenase; TG, triglycerides; TC, total cholesterol; HDL-C, high-density lipoprotein cholesterol; LDL-C, low-density lipoprotein cholesterol; Ca, calcium; GA, glycoalbumin; eGFR, estimated glomerular filtration rate; LPa, lipoprotein(a); sdLDL-C, small dense low-density lipoprotein cholesterol; CysC, cystatin C; BNP, brain natriuretic peptide; AIP, atherogenic index of plasma; UHR, uric acid to high-density lipoprotein cholesterol ratio; NHHR, non-high-density lipoprotein cholesterol to high-density lipoprotein cholesterol ratio; TyG, triglyceride-glucose index; LVEF, Left Ventricular Ejection Fraction.

| **Supplementary Table S3. Baseline Characteristics of the Validation Set** | | | | |
| --- | --- | --- | --- | --- |
|  |  |  |  |  |
| Variables | Total(n=820) | No composite outcome(n=543) | Composite outcome(n=277) | *p* |
| HFpEF(%) | 530 (64.63) | 343 (63.17) | 187 (67.51) |  |
| HFrEF(%) | 290(29.27) | 200 (36.83) | 90 (32.49) |  |
| LVEF (median [IQR]) | 58.00 [35.00, 64.00] | 58.00 [35.00, 64.00] | 59.00 [37.00, 65.00] | 0.081 |
| Causes of heart failure, n (%) |  |  |  |  |
| Ischaemic cardiomyopathy (%) | 336 (40.93) | 202 (37.20) | 134 (48.20) | 0.003 |
| Valvular heart disease (%) | 180 (21.92) | 128 (23.57) | 52 (18.71) | 0.132 |
| Dilated cardiomyopathy (%) | 144 (17.54) | 88 (16.21) | 56 (20.14) | 0.191 |
| Hypertensive heart disease (%) | 132 (16.08) | 98 (18.05) | 34 (12.23) | 0.041 |
| Other (%) | 24 ( 2.92) | 19 ( 3.50) | 5 ( 1.80) | 0.25 |
| age (median [IQR]) | 61.00 [52.00, 70.00] | 62.00 [52.00, 70.00] | 60.00 [53.00, 69.00] | 0.598 |
| BMI (median [IQR]) | 25.00 [22.90, 27.20] | 24.60 [22.70, 26.90] | 25.40 [23.30, 27.70] | 0.005 |
| sex (%) |  |  |  | 0.971 |
| Female | 236 (28.78) | 157 (28.91) | 79 (28.52) |  |
| Male | 584 (71.22) | 386 (71.09) | 198 (71.48) |  |
| hypertension (%) | 404 (49.27) | 235 (43.28) | 169 (61.01) | <0.001 |
| diabetes (%) | 292 (35.61) | 173 (31.86) | 119 (42.96) | 0.002 |
| hyperlipidemia (%) | 419 (51.10) | 246 (45.30) | 173 (62.45) | <0.001 |
| Cr (median [IQR])，(μmol/L) | 80.65 [64.40, 98.82] | 79.80 [64.20, 98.15] | 81.40 [66.10, 98.90] | 0.505 |
| PA (median [IQR]), (g/L) | 0.19 [0.11, 0.25] | 0.19 [0.11, 0.24] | 0.20 [0.11, 0.26] | 0.1 |
| Hcy (median [IQR])，(μmol/L) | 12.14 [7.94, 16.20] | 11.64 [7.86, 15.71] | 12.88 [8.39, 17.30] | 0.053 |
| hs-CRP (median [IQR])，(mg/L) | 3.80 [0.79, 20.25] | 4.61 [0.80, 40.87] | 2.89 [0.77, 20.00] | 0.332 |
| ALT (median [IQR])，(U/L) | 19.00 [13.00, 29.00] | 19.00 [13.00, 29.00] | 20.00 [13.00, 29.00] | 0.984 |
| AST (median [IQR])，(U/L) | 20.16 [15.36, 28.80] | 20.16 [15.36, 27.84] | 21.32 [15.60, 30.78] | 0.104 |
| Alb (median [IQR])，(g/L) | 41.40 [37.10, 44.90] | 41.40 [37.40, 44.80] | 41.40 [36.30, 45.00] | 0.854 |
| TB (median [IQR])，(μmol/L) | 13.10 [9.05, 19.60] | 13.32 [9.31, 20.14] | 12.70 [8.10, 18.42] | 0.042 |
| DB (median [IQR])，(μmol/L) | 4.10 [2.70, 6.47] | 4.14 [2.80, 6.49] | 4.00 [2.50, 6.44] | 0.178 |
| ALP (median [IQR])，(U/L) | 79.00 [57.00, 122.25] | 78.00 [57.00, 128.50] | 82.00 [57.00, 116.00] | 0.997 |
| GGT (median [IQR])，(U/L) | 39.68 [17.28, 240.96] | 41.28 [16.80, 240.96] | 35.88 [18.72, 247.52] | 0.998 |
| TBA (median [IQR])，(μmol/L) | 4.00 [1.80, 6.73] | 4.10 [1.80, 6.80] | 3.70 [1.93, 6.70] | 0.701 |
| ChE (median [IQR])，(kU/L) | 6.40 [3.90, 8.40] | 6.50 [4.10, 8.45] | 6.40 [3.80, 8.30] | 0.372 |
| Urea (median [IQR])，(mmol/L) | 6.63 [5.17, 9.08] | 6.63 [5.16, 8.80] | 6.65 [5.17, 9.57] | 0.549 |
| UA (median [IQR])，(μmol/L) | 321.23 [248.21, 412.58] | 310.46 [237.17, 398.00] | 351.44 [267.44, 449.40] | <0.001 |
| Glu (median [IQR])，(mmol/L) | 5.69 [4.87, 7.23] | 5.71 [4.87, 7.25] | 5.69 [4.84, 7.20] | 0.535 |
| CK (median [IQR])，(U/L) | 63.00 [39.00, 104.10] | 63.00 [40.00, 104.05] | 65.00 [37.10, 105.00] | 0.746 |
| LD (median [IQR])，(U/L) | 186.00 [152.00, 230.00] | 187.00 [153.50, 231.45] | 184.00 [151.00, 228.00] | 0.283 |
| TG (median [IQR])，(mmol/L) | 1.35 [0.84, 2.15] | 1.29 [0.83, 2.15] | 1.40 [0.86, 2.15] | 0.416 |
| TC (median [IQR])，(mmol/L) | 3.39 [2.39, 4.30] | 3.42 [2.48, 4.39] | 3.36 [2.29, 4.20] | 0.208 |
| HDL-C (median [IQR])，(mmol/L) | 1.02 [0.72, 1.24] | 1.05 [0.76, 1.28] | 0.94 [0.69, 1.11] | <0.001 |
| LDL-C (median [IQR])，(mmol/L) | 1.78 [0.95, 2.63] | 1.81 [0.97, 2.65] | 1.76 [0.95, 2.62] | 0.643 |
| Ca (median [IQR])，(mmol/L) | 2.30 [2.09, 2.41] | 2.29 [2.09, 2.41] | 2.31 [2.10, 2.41] | 0.992 |
| GA (median [IQR])，(g/dl) | 13.35 [9.95, 15.38] | 12.91 [9.87, 14.98] | 14.20 [10.90, 16.07] | <0.001 |
| eGFR (median [IQR])，(mL/min/1.73m^2) | 81.10 [57.48, 96.12] | 81.55 [59.65, 96.14] | 80.27 [50.93, 95.92] | 0.206 |
| LPa (median [IQR])，nmol/L | 24.15 [8.57, 67.17] | 24.10 [8.35, 68.00] | 24.60 [8.80, 65.00] | 0.936 |
| sdLDL-C (median [IQR])，mmol/L | 0.19 [0.06, 0.50] | 0.18 [0.06, 0.48] | 0.22 [0.06, 0.60] | 0.004 |
| CysC (median [IQR])，mg/L | 1.17 [0.79, 1.55] | 1.15 [0.77, 1.49] | 1.21 [0.85, 1.71] | 0.025 |
| BNP (median [IQR])，pg/ml | 158.50 [49.00, 499.25] | 175.00 [52.00, 474.50] | 149.00 [40.00, 531.00] | 0.438 |
| AIP (median [IQR]) | 1.17 [0.74, 1.71] | 1.13 [0.69, 1.66] | 1.27 [0.86, 1.86] | 0.003 |
| UHR (median [IQR]) | 0.14 [0.10, 0.23] | 0.13 [0.09, 0.20] | 0.16 [0.12, 0.26] | <0.001 |
| NHHR (median [IQR]) | 2.43 [1.75, 3.41] | 2.36 [1.69, 3.30] | 2.56 [1.88, 3.59] | 0.022 |
| TyG (median [IQR]) | 4.70 [4.44, 4.99] | 4.69 [4.44, 5.02] | 4.75 [4.44, 4.98] | 0.558 |

Abbreviations: IQR, interquartile range; HFrEF, Heart Failure with reduced Ejection Fraction; HFpEF, Heart Failure with preserved Ejection Fraction; BMI, body mass index; Cr, creatinine; PA, prealbumin; Hcy, homocysteine; hs-CRP, high-sensitivity C-reactive protein; ALT, alanine aminotransferase; AST, aspartate aminotransferase; Alb, albumin; TB, total bilirubin; DB, direct bilirubin; ALP, alkaline phosphatase; GGT, gamma-glutamyl transferase; TBA, total bile acid; ChE, cholinesterase; UA, uric acid; Glu, glucose; CK, creatine kinase; LD, lactate dehydrogenase; TG, triglycerides; TC, total cholesterol; HDL-C, high-density lipoprotein cholesterol; LDL-C, low-density lipoprotein cholesterol; Ca, calcium; GA, glycoalbumin; eGFR, estimated glomerular filtration rate; LPa, lipoprotein(a); sdLDL-C, small dense low-density lipoprotein cholesterol; CysC, cystatin C; BNP, brain natriuretic peptide; AIP, atherogenic index of plasma; UHR, uric acid to high-density lipoprotein cholesterol ratio; NHHR, non-high-density lipoprotein cholesterol to high-density lipoprotein cholesterol ratio; TyG, triglyceride-glucose index; LVEF, Left Ventricular Ejection Fraction.

**Supplementary Table S4. Comparison of AUC values and DeLong test results among UHR and other composite lipid indices**

| Group | Comparison | AUC difference(95％CI) | P.value | P.value(adjust) |
| --- | --- | --- | --- | --- |
| Overall | UHR vs AIP | 0.620 (0.039-0.092) | ＜0.001 | ＜0.001 |
| Overall | UHR vs NHHR | 0.620 (0.056-0.106) | ＜0.001 | ＜0.001 |
| Overall | UHR vs TyG | 0.620 (0.066-0.131) | ＜0.001 | ＜0.001 |
| HFrEF | UHR vs AIP | 0.636 (0.036-0.128) | ＜0.001 | ＜0.001 |
| HFrEF | UHR vs NHHR | 0.636 (0.016-0.104) | 0.008 | 0.024 |
| HFrEF | UHR vs TyG | 0.636(0.072-0.186) | ＜0.001 | ＜0.001 |
| HFpEF | UHR vs AIP | 0.617(0.030-0.094) | ＜0.001 | ＜0.001 |
| HFpEF | UHR vs NHHR | 0.617 (0.061-0.122) | ＜0.001 | ＜0.001 |
| HFpEF | UHR vs TyG | 0.617 (0.041-0.127) | ＜0.001 | ＜0.001 |

Abbreviations: UHR, uric acid-to-high-density lipoprotein cholesterol ratio; AIP, atherogenic index of plasma; NHHR, non-high-density lipoprotein cholesterol to high-density lipoprotein cholesterol ratio; TyG, triglyceride-glucose index; AUC, area under the receiver operating characteristic curve; CI, confidence interval. P values derived from DeLong test; adjusted P values calculated using Bonferroni correction for three comparisons within each subgroup.

| **Supplementary Table S5. Predictive performance of UHR and other biomarkers for the 180‑day composite outcome** | | | | | | | |
| --- | --- | --- | --- | --- | --- | --- | --- |
|  |  |  |  |  |  |  |  |
| Indicator | AUC (95% CI) | Best Cutoff | Youden Index | Sensitivity | Specificity | PPV | NPV |
| UHR | 0.620 (0.599-0.642) | 0.134 | 0.209 | 0.703 | 0.506 | 0.421 | 0.769 |
| Cr | 0.526 (0.503-0.548) | 67.45 | 0.058 | 0.744 | 0.314 | 0.357 | 0.706 |
| Hcy | 0.556 (0.533-0.579) | 12.64 | 0.116 | 0.528 | 0.588 | 0.396 | 0.709 |
| CK | 0.514 (0.492-0.537) | 88.5 | 0.031 | 0.695 | 0.335 | 0.348 | 0.683 |
| eGFR | 0.493 (0.47-0.515) | 71.11 | 0.02 | 0.625 | 0.394 | 0.345 | 0.673 |
| BNP | 0.509 (0.486-0.533) | 954.5 | 0.048 | 0.152 | 0.896 | 0.429 | 0.674 |
| CysC | 0.555 (0.532-0.578) | 1.06 | 0.11 | 0.652 | 0.458 | 0.381 | 0.72 |
| hs-CRP | 0.506 (0.484-0.529) | 13.365 | 0.036 | 0.64 | 0.396 | 0.352 | 0.683 |

Abbreviations: UHR, uric acid to high‑density lipoprotein cholesterol ratio; Cr, creatinine; Hcy, homocysteine; CK, creatine kinase; eGFR, estimated glomerular filtration rate; BNP, brain natriuretic peptide; CysC, cystatin C; hs‑CRP, high‑sensitivity C‑reactive protein; CI, confidence interval; PPV, positive predictive value; NPV, negative predictive value.

| **Supplementary Table S6. Multivariable Logistic Regression Analysis for Factors Associated with the Composite Outcome** | | | | | | | |
| --- | --- | --- | --- | --- | --- | --- | --- |
|  |  |  |  |  |  |  |  |
| Variable | Beta | SE | Wald | OR | Lower_CI | Upper_CI | p_value |
| diabetes | 0.076 | 0.114 | 0.669 | 1.079 | 0.863 | 1.349 | 0.503 |
| hyperlipidemia | 0.485 | 0.106 | 4.58 | 1.624 | 1.32 | 1.999 | ≤0.001 |
| BMI | 0.074 | 0.015 | 5.005 | 1.077 | 1.046 | 1.108 | ≤0.001 |
| Cr | -0.004 | 0.001 | -2.41 | 0.996 | 0.994 | 0.999 | 0.016 |
| Hcy | 0.015 | 0.006 | 2.287 | 1.015 | 1.002 | 1.028 | 0.022 |
| AST | 0.001 | 0.001 | 0.731 | 1.001 | 0.999 | 1.002 | 0.465 |
| Alb | 0.013 | 0.007 | 1.947 | 1.013 | 1 | 1.026 | 0.052 |
| GGT | 0.001 | 0 | 2.349 | 1.001 | 1 | 1.001 | 0.019 |
| TBA | 0.001 | 0.003 | 0.284 | 1.001 | 0.995 | 1.007 | 0.776 |
| Glu | -0.058 | 0.022 | -2.587 | 0.944 | 0.903 | 0.986 | 0.01 |
| TC | -0.224 | 0.051 | -4.423 | 0.799 | 0.723 | 0.883 | ≤0.001 |
| GA | 0.068 | 0.012 | 5.538 | 1.07 | 1.045 | 1.096 | ≤0.001 |
| sdLDL-C | 0.7 | 0.169 | 4.146 | 2.013 | 1.446 | 2.802 | ≤0.001 |
| CysC | 0.262 | 0.091 | 2.881 | 1.3 | 1.088 | 1.554 | 0.004 |
| AIP | 0.074 | 0.081 | 0.913 | 1.077 | 0.919 | 1.261 | 0.361 |
| UHR | 1.387 | 0.319 | 4.349 | 4.004 | 2.143 | 7.481 | ≤0.001 |

Abbreviations: SE, standard error; OR, odds ratio; CI, confidence interval; BMI, body mass index; Cr, creatinine; Hcy, homocysteine; AST, aspartate aminotransferase; Alb, albumin; GGT, gamma-glutamyl transferase; TBA, total bile acid; Glu, glucose; TC, total cholesterol; GA, glycoalbumin; sdLDL-C, small dense low-density lipoprotein cholesterol; CysC, cystatin C; AIP, atherogenic index of plasma; UHR, uric acid to high-density lipoprotein cholesterol ratio.

| **Supplementary Table S7. Variance Inflation Factor test** | |
| --- | --- |
| Variable | VIF |
| hyperlipidemia | 1.011 |
| BMI | 1.003 |
| Hcy | 1.202 |
| GA | 1.357 |
| sdLDL-C | 1.13 |
| UHR | 1.114 |

Abbreviations:UHR, uric acid to high-density lipoprotein cholesterol ratio; GA, glycoalbumin; sdLDL-C, small dense low-density lipoprotein cholesterol; BMI, body mass index; Hcy, homocysteine.

| **Supplementary Table S8. Hyperparameter Configurations of the Final Machine Learning Models** | |
| --- | --- |
|  |  |
| Machine learning models | parameter configuration |
| DT | {'ccp_alpha': 0.0, 'max_depth': 10, 'max_features': None, 'min_samples_split': 20} |
| RF | n_estimators = 450 , max_features = 2 |
| XGBoost | {'learning_rate': 0.2, 'max_depth': 3, 'n_estimators': 50, 'subsample': 1.0} |
| LightGBM | {'colsample_bytree': 0.6, 'learning_rate': 0.1, 'n_estimators': 100, 'num_leaves': 31, 'subsample': 0.6} |
| SVM | {'C': 0.1, 'degree': 2, 'gamma': 'scale', 'kernel': 'linear'} |
| ANN | {'activation': 'tanh', 'hidden_layer_sizes': (50, 50)} |

Abbreviations: DT, Decision Tree; RF, Random Forest; XGBoost, Extreme Gradient Boosting; LightGBM, Light Gradient Boosting Machine; SVM, Support Vector Machine; ANN, Artificial Neural Network.
